# Soft Mechanical Inductive Sensors: Principles, Design, and Applications

**DOI:** 10.3390/mi17091051

**Published:** 2026-09-02

**Authors:** Muhammad Awais, Gayatri Indukumar, Diana Cafiso, Lucia Beccai

**Affiliations:** 1 Soft BioRobotics Perception Laboratory, Istituto Italiano di Tecnologia, 16163 Genova, Italy; gayatri.indukumar@iit.it (G.I.); diana.cafiso@iit.it (D.C.); 2 Biorobotics Institute, Scuola Superiore Sant’Anna, 56025 Pontedera, Italy; 3 Department of Computer Science, Bioengineering, Robotics, and Systems Engineering—DIBRIS, University of Genova, 16145 Genova, Italy

**Keywords:** soft mechanical inductive sensors, proprioception, angle sensors, coil design, printed circuit board (PCB) fabrication, soft lithography, complementary metal-oxide-semiconductor (CMOS) fabrication, soft robotics, biomedical applications

## Abstract

Soft mechanical inductive (SMI) sensors are emerging as a promising solution for advanced robotics, healthcare, and wearable devices, offering high precision, adaptability, and environmental robustness. These sensors leverage coil-based designs to achieve resilience against temperature variations, humidity, and mechanical wear, making them suitable for long-term operation in challenging environments. Existing reviews tend to focus narrowly on specific applications of coil-based inductive sensors, such as soft-robotic tactile sensing or biomedical devices, without systematically comparing design methodologies, fabrication techniques, or broader use cases, thereby lacking a unified perspective on the field. This review addresses this gap by analyzing recent developments in SMI sensors from theoretical concepts and practical design to their use cases. The review focuses on the working principles, design strategies, fabrication techniques, electronic interfaces, and, finally, the applications of coil-based SMI sensors. Key applications in soft robotics, prosthetics, and haptic devices are examined, highlighting the transformative potential of these sensors across diverse domains. Finally, the review discusses critical challenges, including sensitivity optimization, durability, and environmental interference, and outlines future directions to further advance this promising technology.

## 1. Introduction

Mechanical sensing provides information from physical stimuli, allowing systems in robotics [1,2,3] and healthcare [4,5,6,7,8,9,10,11] to perceive their own state and tactile cues, including deformation, contact forces, pressure distributions, and surface characteristics [12,13,14].

Numerous sensing approaches have been developed, including piezoresistive, piezoelectric, capacitive, and optical modalities [15,16,17,18,19]. Although each technology offers unique benefits, they also present inherent trade-offs in sensitivity, dynamic range, spatial resolution, hysteresis, mechanical durability, and cost. Comprehensive reviews of other transduction methods are available for piezoresistive [12], piezoelectric [20], capacitive [21], and optical sensing [22,23]. Depending on their geometry, target material and configuration, operating frequency, mechanical construction, and readout electronics, inductive sensors can exhibit high linearity, low hysteresis, and good repeatability [24,25,26]. They may also be less susceptible to electric-field and proximity interference than unshielded capacitive sensors [27] and can provide greater mechanical robustness than some vision-based optical tactile sensors, particularly when interacting with hard, rough, or sharp objects [24,28]. Table 1 provides a comparison of core performance indicators for various sensing mechanisms. The reported sensitivity values are device-specific, illustrative examples expressed in mechanism-dependent units rather than direct comparative benchmarks. These advantages are therefore application and implementation dependent rather than universal characteristics of the sensing principle [29].

In general, inductive sensing provides high-resolution measurements under both static and dynamic conditions [30], excellent durability in harsh environments, including underwater, contaminated, and construction-site settings [24,31,32], and immunity to environmental disturbances [24,33]. The sensing mechanism relies on low-cost components, such as spiral-shaped coils fabricated from conductive traces, threads, or wires. For tactile sensing applications, these coils are integrated with mold-cast or three-dimensional (3D)-printed elastomeric structures that serve as mechanical modulation layers and are coupled with conductive or ferromagnetic target layers. As discussed throughout this review, key sensing characteristics can be readily tuned by modifying different aspects of the sensor design, making inductive sensors versatile and promising candidates for soft robotic and wearable applications. Accordingly, the scope of this paper encompasses coil-based sensors operating according to inductive sensing principles governed by Faraday’s law in which the coil, the target structure, or both are soft or mechanically compliant. This class of devices is collectively referred to herein as “soft mechanical inductive sensors”.

Although soft inductive sensing technologies have drawn significant research interest, existing review articles address the topic only partially. Most of the literature confines inductive sensors to brief subsections: ref. [29] touches upon soft-robotic tactile sensing, ref. [34] outlines robotic tactile arrays without offering detailed design or manufacturing comparisons across application regimes, and [35] focuses narrowly on 3D-printed metamaterial architecture-based inductive sensors. The sole dedicated review on inductive sensors [36] is tailored specifically to healthcare applications, offering only a superficial overview of fabrication methods. Consequently, a comprehensive review encompassing the complete spectrum of SMI sensor design, systematic fabrication comparisons, and multi-domain applications remains absent from the literature. To the best of our knowledge, currently, there is no comprehensive review that covers all aspects of SMI sensor design in addition to fabrication methodologies and applications. This resource is crucial for the scientific community to understand and systematically design innovative inductive sensors for a wide range of target applications.

**Figure 1:**
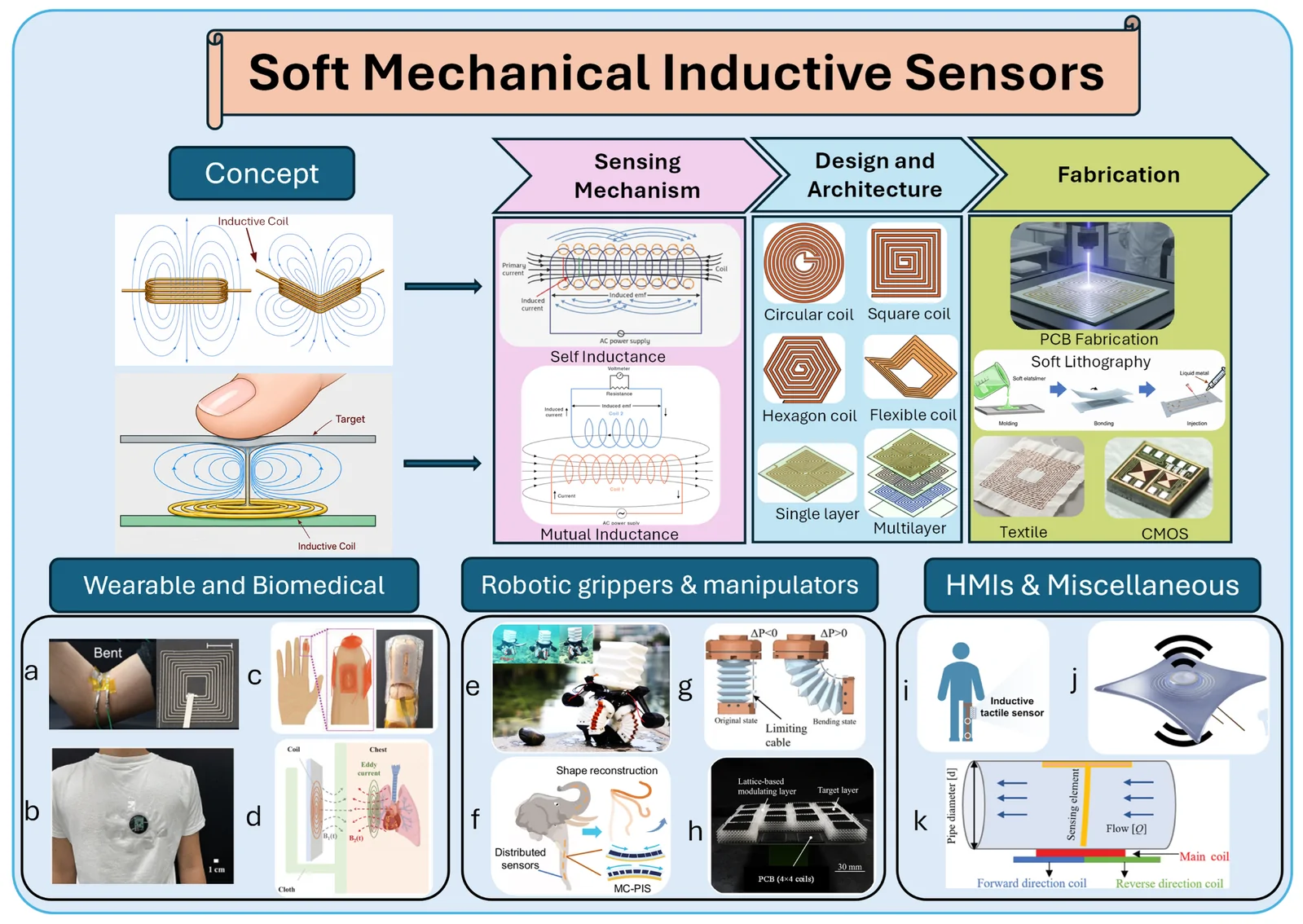
Comprehensive overview of SMI sensors, illustrating their evolution from fundamental sensing principles to advanced applications across multiple domains. The upper panel presents the underlying sensing mechanisms based on self-inductance and mutual inductance together with representative sensor architectures. It also highlights key design approaches, such as PCB manufacturing, soft lithography, textile integration, and complementary metal-oxide-semiconductor (CMOS) fabrication, which facilitate the development of flexible and stretchable inductive sensing platforms. The lower panel summarizes state-of-the-art applications in the following: (i) wearable and biomedical systems, including (**a**) a square liquid metal coil attached to the inner side of the elbow to monitor the angle (scale bar: 10 mm). Reproduced with permission from Ref. [37]. Copyright (2020) Wiley, (**b**) a pocket-size cardiopulmonary sensor. Reproduced with permission from Ref. [38]. Copyright (2024) IEEE, (**c**) a finger-mounted inductive array for tactile stimulus localization. Reproduced with permission from Ref. [39]. Copyright (2024) IEEE, and (**d**) cardiopulmonary monitoring. Reproduced with permission from Ref. [38]. Copyright (2024) IEEE; (ii) robotic grippers and manipulation, including (**e**) an intelligent origami jellyfish robot. Reproduced with permission from Ref. [40]. Copyright (2022) Wiley, (**f**) illustration of shape sensing of an elephant-inspired soft continuum robot. Reproduced with permission from Ref. [41]. Copyright (2025) Nature, (**g**) configuration of origami modules and reconfiguration between linear and bending modules. Reproduced with permission from Ref. [40]. Copyright (2022) Wiley, and (**h**) a surface-based manipulation platform. Reproduced with permission from Ref. [42]. Copyright (2026) IEEE; and (iii) human–machine interface and miscellaneous applications, including (**i**) a tactile sensor integrated between the leg and a reciprocating gait orthosis (RGO). Reproduced with permission from Ref. [30]. Copyright (2025) OAE Publishing Inc., (**j**) a stretchable acoustic device (SAD) functioning as a loudspeaker. Reproduced with permission from Ref. [43]. Copyright (2015) Springer Nature, and (**k**) a water-flow sensing system. Reproduced with permission from Ref. [44]. Copyright (2024) IEEE.

To address this gap, this review covers the fundamental principles, design, fabrication, and applications of SMI sensors in soft robotics, wearable systems, and human–machine interaction (Figure 1). This work is organized as follows: Section 1 introduces the fundamentals of inductive sensors, which are followed by the design, architecture, and electronic readout systems. Subsequently, various fabrication techniques for inductive sensors are discussed in detail. Section 7 then provides in-depth insight into the use of inductive sensors in robotics and soft robotics, wearable and biomedical systems, and wireless-based approaches used in those fields. Finally, the current limitations and future directions of SMI sensors are discussed.

To ensure comprehensive coverage of recent advances in SMI sensors, a literature search was conducted across major scientific databases, including IEEE Xplore, Scopus, Web of Science, ScienceDirect, and Google Scholar, for publications up to 2026. Keywords included “soft inductive sensor,” “inductive tactile sensor,” “flexible/planar inductive sensor,” “soft robotics,” and “soft sensor fabrication.” Retrieved publications were screened by title, abstract, and full text, and the review papers were included if the following criteria were met: (i) presented the design, fabrication, modeling, characterization, or application of soft or flexible inductive sensors; (ii) focused on or featured inductive sensing; (iii) appeared in peer-reviewed journals or conference proceedings; and (iv) were written in English.

**Table 1:** Representative performance comparison of reported tactile sensing mechanisms.

Mechanism	Sensitivity	Hysteresis	Environmental Robustness	Cost	Disadvantage	Ref.
Piezoresistive	174.83 kPa^−1^ (10 Pa to 500 Pa)	0.8%	High	Low	Affected by temperature and mechanical stress	[45]
Piezoelectric	10.53 mV N^−1^	5.88%	High	High	Affected by external vibration	[46]
Capacitive	0.27632 N^−1^ (normal) 0.14634 N^−1^ (tangential)	3.5% (normal) 5.1% (tangential)	High	High	Susceptible to electromagnetic interference	[47]
Optical	—	5.45%	Low	High	Affected by ambient light	[23]
Inductive	39.04 μH N^−1^	8.7%	Very High	Low	Susceptible to magnetic and electric field interference	[42]

Note: Sensitivity values are device-specific metrics expressed in mechanism-dependent units under varying testing conditions; they serve as illustrative examples rather than direct quantitative benchmarks.

## 2. Fundamentals of Inductive Sensing

Inductive sensors are based on electromagnetic induction [48], in which an alternating current (AC) current through a coil produces an oscillating magnetic field. The presence or motion of a conductive or ferromagnetic target perturbs this field, leading to measurable changes in coil inductance or energy dissipation (Figure 1). Conventional inductive sensing systems generally comprise three essential elements: a sensing coil that generates the magnetic field, an excitation and readout circuit that drives the coil and monitors its electrical response, and a conductive or ferromagnetic target that interacts with the magnetic field. When the target approaches or moves relative to the coil, eddy currents or magnetic field perturbations are produced, resulting in measurable changes in inductance or energy dissipation. While traditional inductive sensors are primarily employed for non-contact proximity and displacement sensing of metallic objects, the same transduction mechanism can be extended to soft mechanical sensors. By incorporating deformable structures that modulate the relative position between the coil and target under external stimuli, inductive sensing can be utilized to measure force, pressure, strain, and other tactile inputs (Figure 1). The guiding principle behind the design of an inductive sensor is Faraday’s laws of induction, which define the relationship between the time-varying magnetic field B of a coil and the induced voltage E. The generalized equation is as follows:

(1) $$E=\oint_{C}E\;\cdot \;dl=-\frac{d}{dt}\int_{S}B\;\cdot \;dS,$$

where *C* stands for any closed conducting loop (arbitrary in shape and size), *S* is any surface bounded by *C*, and E is the electric field. Variations in the amplitude, frequency, or phase of the resulting signal can be correlated with displacement, force, or shape change.

This fundamental principle can be exploited in several ways, depending on the number of coils or the chosen target:

- In self-inductance sensors, the presence of a target directly alters the inductance of a single coil through changes in magnetic permeability or energy loss [26,49,50]. They are primarily used for uniaxial force and pressure sensing and for measuring proximity by monitoring changes in the inductance of their own coils.
- By employing two or more coils arranged in proximity, one can make use of the phenomenon of mutual inductance. In this case, one coil serves as a transmitter and the other as a receiver. The motion of the target layer alters the magnetic coupling between the coils, leading to measurable voltage changes [51,52,53,54,55]. These are commonly used for the continuous monitoring of non-uniform bending in robotic grippers and compliant actuators.
- Conductive targets use a specific phenomenon known as the eddy current effect [56]. When conductive materials are exposed to an alternating current AC magnetic field, circulating eddy currents are induced, which generate opposing magnetic fields and modify the effective inductance of the coil. This effect enables the precise detection of deformation and force [24,48,57,58,59,60,61].
- Ferromagnetic targets are materials with high magnetic permeability. This implies that the magnetic field produced by the coil concentrates on the target as it approaches it. This reduces the circuit’s net magnetic reluctance and increases the coil’s inductance.

The aforementioned inductive sensing mechanisms can be utilized depending on the application scenario. Self-inductance is commonly used for contact force and pressure sensing, offering a simple, low-cost solution but with limited spatial information. Mutual inductance is suitable for sensing non-uniform bending in robotic grippers and soft actuators, providing high accuracy and a large sensing range at the expense of more complex fabrication and signal processing. Eddy current-based sensors enable non-contact displacement sensing with high sensitivity, wide bandwidth, and robustness to environmental contaminants, but they are limited to conductive targets. Ferromagnetic target-based inductive sensors can be used in magnetic encoders and soft tactile sensors, providing larger inductance variations and a more linear response; however, they are susceptible to interference from permanent magnets and strong external magnetic fields.

This section provides a general overview of the fundamental principles governing this sensing modality. For a more comprehensive treatment of complex impedance, including series resistance, quality factor, skin depth, and self-resonance, readers are referred to [62]. A complementary discussion with a stronger emphasis on industrial applications is provided in [63].

## 3. Design and Architecture

The objective of this section is to provide a comprehensive overview of the research addressed on the design of inductive sensors. As described in Section 2, the inductive tactile sensor has three layers (i.e., coil, deformable layer, target), and each layer plays a specific role in the sensor’s performance. Therefore, in this section, our analysis is articulated among these three parts.

### 3.1. Coil Design

The main goal in designing coils is to maximize and homogenize the magnetic field they produce while accounting for application-specific design constraints. The performance of inductive sensors is strongly influenced by the design and architecture of the conducting loop of the coil and its enclosed surface area, *S* [64]. This governs the magnetic field distribution and integration potential. The resultant inductance for a generalized coil is given as follows:

(2) $$L=\frac{N}{I}\int_{S}B\left(I\right)\;\cdot \;dS,$$

where *N* is the number of turns of the coil and *I* is the exciting AC current. In addition, the magnetic field generated by the coil is governed by the Biot–Savart law [30]:

(3) $$B=\frac{\mu_{0}}{4\pi }\int_{C}\frac{I\;dl\times \hat{r}}{r^{2}},$$

where dl is the differential length element of the coil path *C*, $\mu_{0}$ is the permeability of free space, *r* is the distance between the coil element and the point under consideration, and $\hat{r}$ is the unit vector pointing toward the point under consideration. From the governing equations, the coil geometry is crucial to its design. This defines a fairly extensive design space for investigation. Furthermore, two main design features must be considered: shape (e.g., square, circular, elliptical) and configuration (i.e., their arrangement in the 3D space). These features have been extensively studied, and the results are summarized below.

#### 3.1.1. Shape

**Figure 2:**
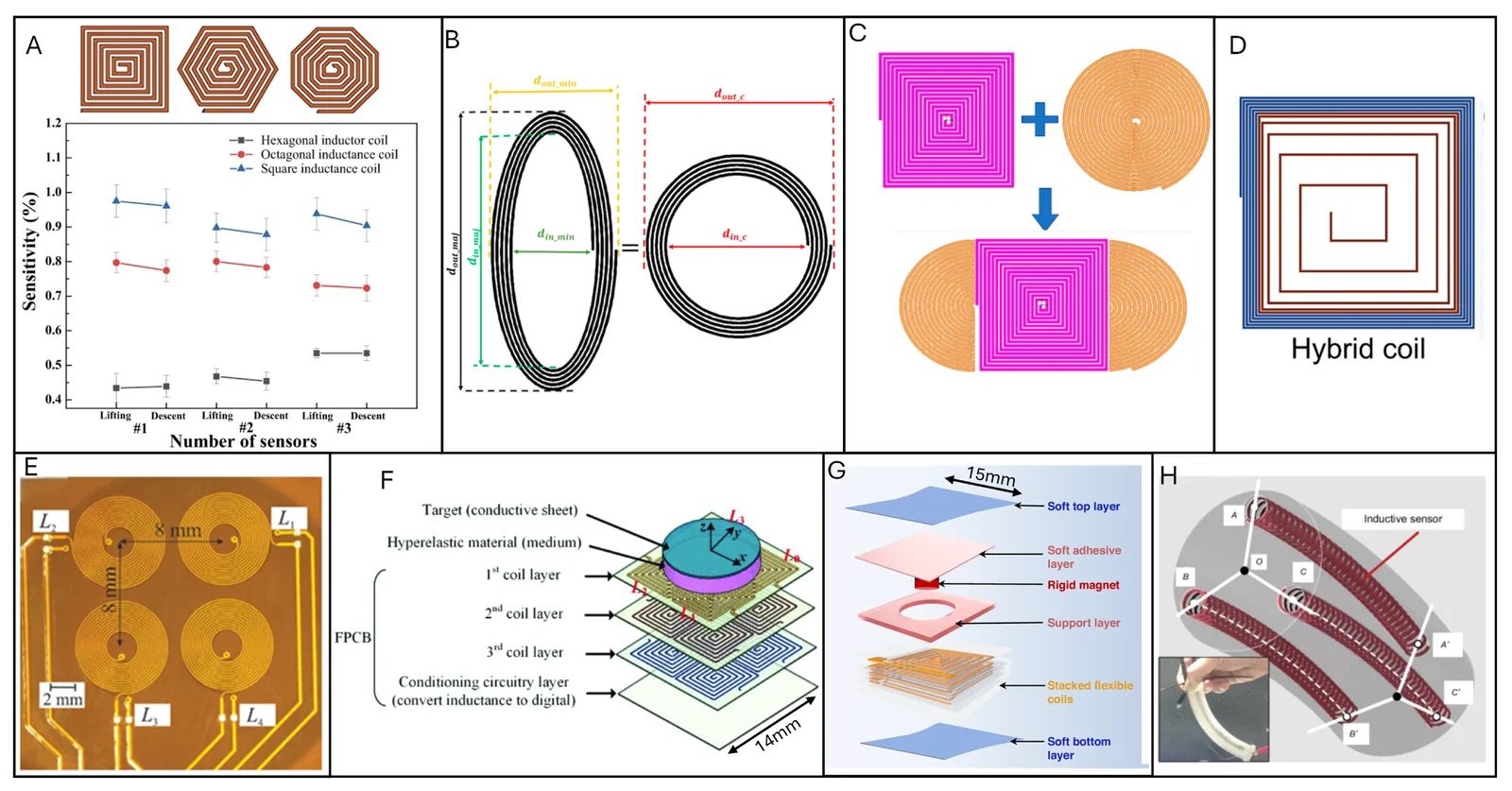
Design strategies of SMI sensors. (**A**) Schematic illustration and sensitivity comparison of 2D coils with different geometries. Reproduced with permission from Ref. [54]. Copyright (2022) Springer Nature. (**B**) Elliptical planar inductor transformation into the circular planar inductor. Reproduced with permission from Ref. [70]. Copyright (2023) IEEE. (**C**) Oval shape design obtained by combining square and circular coils. Reproduced with permission from Ref. [72]. Copyright (2025) MDPI. (**D**) Hybrid coil design featuring a graded density distribution with lower turn density at the center and higher density toward the edges. Reproduced with permission from Ref. [30]. Copyright (2025) OAE Publishing Inc. (**E**) Photograph of single-layer flexible coils. Reproduced with permission from Ref. [33]. Copyright (2018) IEEE. (**F**) Schematic representation of a multilayer flexible sensor comprising embedded coils, deformable dielectric layers, and target layers. Reproduced with permission from Ref. [82]. Copyright (2019) IEEE. (**G**) Multilayer structure of a flexible wide-range multidimensional force sensor. Reproduced with permission from Ref. [83]. Copyright (2024) Springer Nature. (**H**) 3D fiber-reinforced inductive sensing structures and their integration into a soft manipulator prototype. Reproduced with permission from Ref. [26]. Copyright (2020) IEEE.

The most commonly used coil geometries are square [54,65,66,67], hexagonal and octagonal [68], circular [69], and elliptical [70]. Several studies have systematically compared the influence of coil shape on various parameters [54,71,72]. Overall, increasing the number of longer parallel straight segments (e.g., square geometry vs. circular or hexagonal) improves sensitivity to bending and folding [54] deformation (as shown in Figure 2A, approximately 1.5× that of the octagon and 2.4× that of the hexagon). Moreover, Wang et al. [66] also analyzed various shapes of the coils made of liquid metal, including rectangular, circular, elliptical, and square, and they concluded that the aspect ratio (AR) is the critical parameter which affects the planar coil’s sensitivity to strain. Farooq et al. [70] presented a computationally efficient model for space-constrained elliptical planar inductors by transforming their parameters into equivalent circular geometries (Figure 2B), achieving high-accuracy predictions of inductance and parasitic properties with less than 5% experimental error across 75 fabricated prototypes. Pacurar et al. [72] designed a new oval-shaped inductor and accurately predicted its inductance, as shown in Figure 2C. The proposed planar spiral inductor shape increases inductance by 2.16× compared to square, 1.84× to hexagonal, 2.12× to octagonal, and 2.52× to circular designs.

Beyond comparative studies, application-driven design strongly influences coil selection. For instance, Wang et al. [24] implemented a planar spiral coil on a flexible PCB to maximize the sensing area while maintaining a thin conformal structure suitable for soft substrates, showing potential for real-world applications. By optimizing turn count, trace width, and spacing, they achieved a balance among sensitivity, inductance, and resistance, preserving a high quality factor (QF) that indicates low energy loss. To improve lateral shear sensing performance, Figure 2D shows the design proposed by Chen et al. in [30], a hybrid structure comprising concentric outer turns and spiral inner turns, which are both on the same plane. The results indicate that the design produces a more uniform magnetic field distribution than a conventional, uniformly spaced spiral coil. Table 2 provides a comparative analysis of the key parameters of the different shapes of planar coils.

**Table 2:** Comparison of key performance parameters across various planar coil geometries from different studies.

Geometry	Ref.	*L* Gain ($\Delta L/L_{0}$)	Sensitivity/Specific Metrics	QF
Square	[54]	190%	Bending: ∼0.96%	—
	[30]	Z: 3.5%, Y: 42%	Spatial: Z: ∼1.7%/mm, Y: ∼3.5%/mm	—
	[66]	∼62%	Strain: 0.36	—
Circular	[72]	−14.5%	—	0.138
	[66]	∼42%	Strain: 0.38	—
Hexagonal	[72]	17.2%	—	0.117
Octagon	[72]	1.4%	—	0.067
Oval	[72]	115.7%	—	0.129
Hybrid	[30]	Z: 5.8%, Y: 88%	Bending: 0.0556% °^−1^ Spatial: Z: ∼2.95%/mm, Y: ∼6.33%/mm	—

#### 3.1.2. Configuration

The configuration refers to the arrangement of coils of a given shape in 3D space. Several coil configurations have been explored, including planar, multilayer, and 3D coils. Table 3 summarizes some of them.

The 2D coils can consist of single or multiple layers. Single-layer planar coils are widely used in SMI sensors due to their simplicity, ease of fabrication, and compatibility with flexible substrates [24,30,33,37,73,74,75,76,77,78,79]. Their performance is strongly influenced by the fill ratio (conductor area/total coil area), which determines the winding density and, in turn, the inductance. One such example is shown in Figure 2E, in which Wang et al. [33] proposed a three-axis sensor having four coils (with a fill ratio of 0.18 for each coil) on a flexible substrate. In another study, Wang et al. [37] proposed a planar coil-based bending sensor that uses flexible PCBs with spiral coils to achieve linear ratiometric outputs for cumulative bending measurement in soft robots.

Multilayer coils stack multiple winding layers separated by insulating films, enhancing inductance and magnetic field strength within the same footprint [30,80,81,82,83,84,85,86,87,88]. This architecture improves sensitivity and coupling efficiency, particularly in low-signal environments, although at the cost of increased fabrication complexity and reduced flexibility. A good example can be seen in Figure 2F, which shows a three-layered inductive sensor with all the layers labeled [82]. Interesting developments have been achieved by Kar et al. [81], who designed a bending angle sensor for soft robotic fingers using three stacked flexible PCBs with double-sided spiral coils, achieving linear responses with 0.5° resolution and low hysteresis. Moreover, Zhang et al. [83] developed a flexible wide-range multidimensional force sensor (FWMFS) using multilayer stacked coils (Figure 2G) and an adjustable rigid magnet with sensitivities up to 1.039 mV/N for lateral forces and robust performance over 5000 cycles.

While 2D coils are widely used and are particularly suitable for thin, flexible systems, they limit wireless power transfer (WPT) and coupling efficiency for freeform geometries. The 3D designs, including helical coils [89,90], solenoids [91], and free-form wound geometries [26,68,92,93,94,95,96,97], maximize magnetic flux coupling and provide higher inductance per turn. Figure 2H shows hollow spring coils, which are made of metal and integrated into a soft manipulator to measure the bending angle [26]. In [92], Alghrairi et al. demonstrated enhanced WPT efficiency by combining a single helical receiver coil with three asymmetric rectangular transmitter coils, highlighting the potential of the 3D coil design for efficient energy and signal transfer.

**Table 3:** Comparison of various configurations among coil designs.

Configuration	Pros	Cons	Typical Application	Ref.
Single-Layer Planar	Simplicity and ease of fabrication Compatible with flexible substrates Precise geometries Highly reproducible	Low inductance Low sensitivity Limited WPT Low coupling efficiency	Wearable systems Soft robotics	[24,33,37,73,74,75,76,77,78,79,98]
Multilayer	Higher inductance for a given footprint High sensitivity in low-signal environments	Fabrication complexity Reduced flexibility Limited WPT Mediocre coupling efficiency	Distributed manipulator systems Wireless power transfer Retinal prosthesis application	[30,80,81,82,83,84,85,86,87,88]
3D	Maximizes magnetic flux linkage Moderately easy fabrication Strong coupling and high sensitivity High WPT	Bulky Less compatible with flexible systems	Wireless power transfer Ambulatory monitoring respiration monitoring human motion monitoring	[26,68,92,93,94,95,96]

### 3.2. Deformable Layer

**Figure 3:**
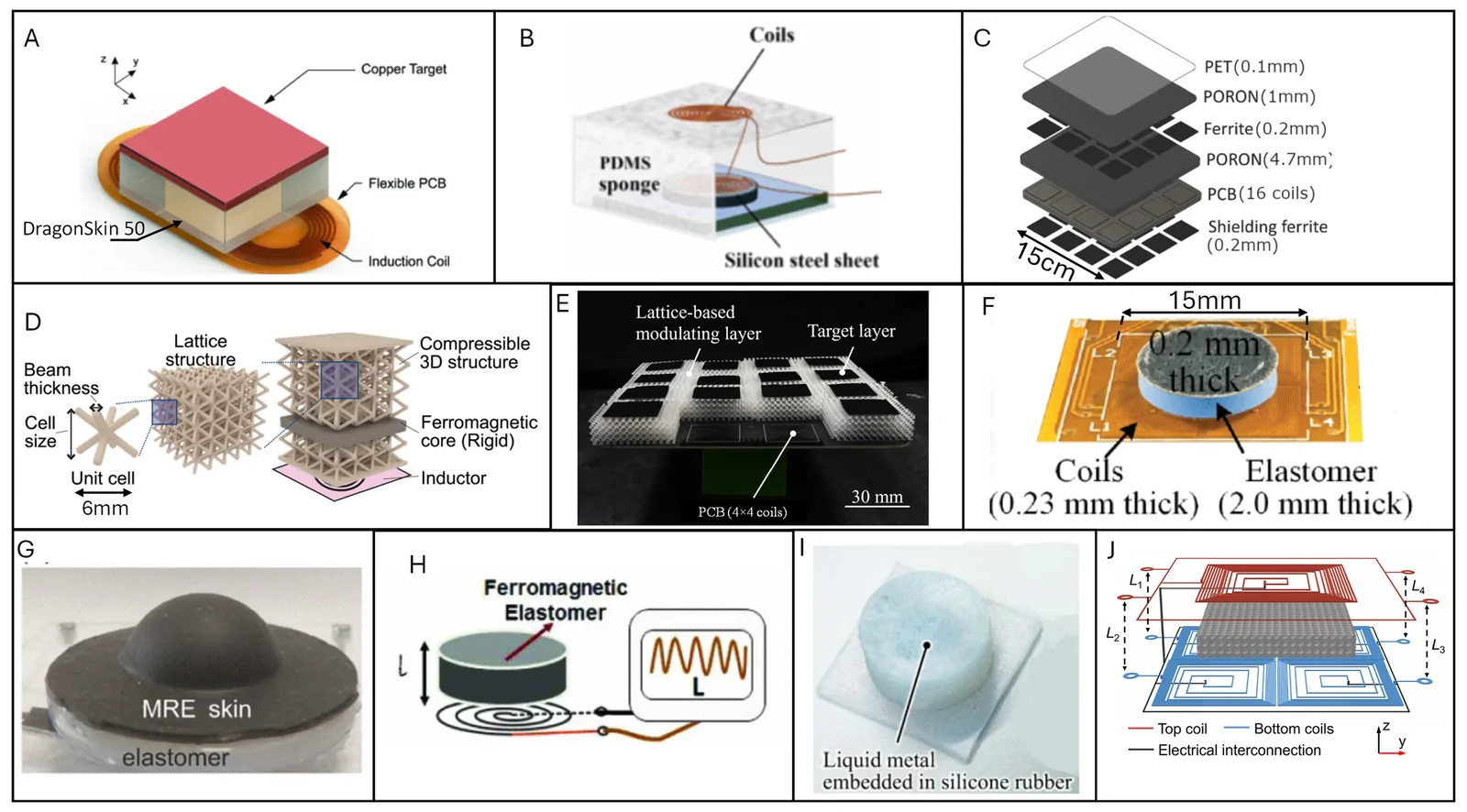
Deformable and target layers: (**A**) A two-axis soft inductive tactile sensor. Two coils are positioned below a copper target and silicone elastomer to detect forces in the z and x axes. Reproduced with permission from Ref. [99]. Copyright (2019) IEEE. (**B**) Schematic of sensor structure by using PDMS as a modulating layer. Reproduced with permission from Ref. [100]. Copyright (2025) IEEE. (**C**) Exploded schematic of a soft sensor prototype using Poron as the deformable layer. Reproduced with permission from Ref. [80]. Copyright (2025) IEEE. (**D**) Schematic structure of a pressure sensor with a deformable layer composed of a 3D-printed lattice structure with an X-cell unit type. Reproduced with permission from Ref. [102]. Copyright (2025) IEEE. (**E**) Photograph of the COPESS-based manipulation setup featuring a deformable layer made of a 3D-printed lattice structure fabricated with soft material (Elastic 50A). Reproduced with permission from Ref. [42]. Copyright (2026) IEEE. (**F**) Three-axis inductive sensor with aluminum as the target layer. Reproduced with permission from Ref. [76]. Copyright (2020) IEEE. (**G**) Photograph of wireless inductive sensors using MRE as both the actuator body and the sensing target. Reproduced with permission from Ref. [104]. Copyright (2019) IEEE. (**H**) Sensor structure incorporating a ferromagnetic elastomer as the target layer. Reproduced with permission from Ref. [105]. Copyright (2019) IEEE. (**I**) Flexible and soft inductive three-axis tactile sensor using liquid metal as the sensing target. Reproduced with permission from Ref. [74]. Copyright (2019) IEEE. (**J**) Schematic of dual three-axis tactile sensors with a hybrid coil as the target. Reproduced with permission from Ref. [30]. Copyright (2025) OAE Publishing Inc.

In SMI sensors, the deformable layer plays a central role in transducing mechanical stimuli into measurable electrical signals. It influences the sensing mechanism by altering the distance and alignment between the coil and the metal target. In terms of design, this layer offers a high degree of freedom and an opportunity to innovate the final sensor design and performance. The most widely used materials are silicone, such as Ecoflex [99] (Figure 3A) [76,99] and polydimethylsiloxane (PDMS) [100] (Figure 3B) [39]. As each coil exhibits an effective sensing region where the electromagnetic interference (EMI) field is sufficiently strong to interact with the target, the thickness of the deformable layer can be optimized to enhance the mechanical sensitivity of inductive sensors. For example, in [101], it was experimentally evaluated that for the given sensor dimensions, the optimal gap *g* is four times the thickness of the magnetic film *t* (g/t=4), at which the prototype’s inductance, inductance ratio, and voltage sensitivity are maximized.

Beyond size, the mechanical properties of the deformable layer are crucial with a softer material achieving higher higher mechanical or electrical sensitivity than a stiffer one at the expense of a lower force measurement range as the sensor achieves saturation quickly [24,39].

The stiffness and the overall mechanical behavior of the deformable layer can be finely controlled by using specifically designed geometries or non-homogeneous materials. In particular, porous materials and lattice architectures emerged as two promising tools to control the inductive sensors’ performance. For instance, ref. [65] reports an example of a striped elastomer as the grooves of the striped elastomer provide space for the soft conductive layer to be compressed to close the planar coil, which significantly improves the force sensing sensitivity, and Figure 3C reports a commercial polyurethane foam to enhance the displacement under compression [80]. Chen et al. [30] developed a porous structure based on an emulsion system that exhibits ordered pores, allowing for adjusting the sensing range from 2 kPa to 3.4 MPa, depending on the pore size. By selecting smaller pore sizes, the sensing range can be extended because the increased structural stiffness of the smaller pores requires a greater external force to reach mechanical deformation and signal saturation. A sensor with 200 μm c-PCL achieves a force range of 0 N to 12.6 N with high sensitivity for both normal and shear force sensing, outperforming comparable inductive sensors [80].

Lattices are geometric designs in which structural elements such as beams, struts, and surfaces are arranged in a repeating pattern throughout a volume. 3D printing, which enables fast prototyping across multiple designs, represents an effective solution to precisely control the design of complex architectures and therefore the performance of a sensor that embeds a lattice structure as the deformable layer. Figure 3D shows a 3D-printed body-centered cubic (body-centered cubic (BCC)) lattice structure made of thermoplastic polyurethane (thermoplastic polyurethane (TPU)), which has been used as a deformable layer [102]. At low pressures, sensors with beam thicknesses of 1.0, 1.2 and 1.4 mm show comparable sensitivities (−0.17, −0.12 and −0.11 kPa^−1^). Above approximately 11, 19 and 30 kPa, inductance saturation occurs, reducing sensitivities to −0.10, −0.06 and −0.03 kPa^−1^, respectively, and exhibiting hysteresis. In a recent study [42], we proposed a 3D-printed gyroidal lattice-based deformable layer for surface-based object manipulation. The lattices are printed using a commercial photo resin ((Elastic 50A Resin, Formlabs Inc., Somerville, MA, USA)) which has good compliance (Shore hardness = 50A) and fast recovery from deformation, as shown in Figure 3E. Our results indicate that softer lattices (relative density of 7%) have 23× higher sensitivity than stiffer ones (relative density of 20%). Moreover, the operational force range increases 9× when increasing the relative density from 7% to 20%. However, limits on cell size (2.2 mm to 4 mm) and relative density (7% to 30%) are imposed by the effective printability of the lattices. At higher relative densities, the material tends to clump, trapping uncured resin in the cell cavities, and if the relative density is lower than 7%, the structure cannot support itself against gravity.

Existing deformable layers split into two design philosophies with complementary trade-offs: porous media rely on stochastic microstructure for simplicity and, in custom variants, multi-axis sensing, while 3D-printed lattices exploit deterministic geometry for tunable control over the sensitivity-range-hysteresis relationship. Commercial polyurethane foam [80] sets a conservative baseline (0.15 N μH^−1^, 0 N to 6.72 N range, <15% hysteresis, stable) but lacks multi-axis capability; the fabricated porous layer [30] adds simultaneous normal (1.11 kPa^−1^) and shear (1.18 kPa^−1^) sensing with low hysteresis (∼5.0% and ∼6.25%) and a wider range (0 N to 12.6 N), demonstrating 1000-cycle stability, at the cost of complex fabrication. Lattices instead diverge into two regimes: the TPU lattice [102] favors elevated pressures (112 kPa to 368 kPa) and is by far the most durable (15% hysteresis over 10,000 cycles), at the expense of accessible low-end sensitivity; the softer Elastic 50A lattice [42] favors higher sensitivity (1.7 μH N^−1^ to 39.04 μH N^−1^) over a broad 0 N to 16.68 N range with moderate hysteresis (8.7%), but its stability has only been shown to 200 cycles, which is an order of magnitude less than the TPU lattice or the fabricated porous layer. No reported design combines high multi-axis sensitivity, wide dynamic range, low hysteresis, and long-term (≥1000-cycle) stability in a single layer.

### 3.3. Target Layer

The choice of the target material depends on the selected sensing mechanism (e.g., eddy currents or magnetic field changes), while specific patterns can unlock new functionalities. The target layer is placed on top of the sensing coil and interacts with the coil’s magnetic field. The interaction between the target layer and the sensing coil is determined by the target’s position, geometry, and electromagnetic properties, which affect the magnetic field distribution and consequently the sensitivity, spatial resolution, mechanical compliance, and robustness of the inductive sensor [103].

- Materials: The target can be conductive or ferromagnetic. Conductive targets usually enable higher sensitivity, while ferromagnetic targets tend to produce a more linear response. Conductive targets are used in sensors that operate by the eddy current principle, commonly copper [24], or aluminum [76] as shown in Figure 3A and Figure 3F, respectively. Ferromagnetic targets modify magnetic permeability and flux concentration, thereby increasing the coil’s overall inductance. They are usually made of ferrite [80].
- Physical integration: Another aspect to consider is how metallic targets, whether conductive or ferromagnetic, are integrated in the sensor. In some cases, they are distinct layers attached to the deformable layer (e.g., via glue). For instance, Dacre et al. [80] made a ferromagnetic target out of ferrite sheets (0.2 mm thickness) that were placed on top of a foam layer. In this case, the thickness was used to maximize compliance and sensitivity. Nevertheless, metallic targets are usually stiffer than the deformable layer, thereby reducing the overall sensor’s compliance. Moreover, the adhesion between completely different materials can cause delamination. This is why magnetorheological elastomers (magnetorheological elastomers (MREs)) (i.e., elastomers including ferromagnetic fillers) [67,75,98] have also been explored as target materials. In Figure 3G, Wang et al. [104] utilize highly stretchable MREs, serving both as the skin of a soft actuator and the target of the sensing system. Similarly, Ozioko et al. [105] incorporated iron into a silicone matrix with a higher content of iron particles that yields better sensitivity and linearity (Figure 3H).
  Instead of relying on a post-assembled conductive target, Figure 3I presents a design by [74], in which liquid metal is employed directly as the sensing target in a three-axis inductive tactile sensor. This approach achieves a 1.7 × higher signal-to-noise ratio, along with sensitivity improvements of 6.6 × in shear and 4.2 × in the normal direction, compared to MRE-based inductive sensors. Alternatively, rather than introducing an additional material layer, the coil itself can be utilized as the target. For example, the design by Chen et al. [30], shown in Figure 3J, employs the same coil structure for both sensing and target functions.
- Position: Maximum inductance variation occurs when the target fully overlaps a single coil. However, when the target moves across the center of the region shared by multiple planar coils, it can produce comparable sensitivity along all three axes [33].

The selection of target layers involves balancing electromagnetic coupling against mechanical compliance and fabrication effort, as demonstrated across target configurations in the literature. Target implementations can broadly be categorized into rigid and soft functional structures. Among rigid schemes, standard conductive sheets (e.g., copper or aluminum) provide baseline inductive targeting with minimal fabrication complexity but zero mechanical compliance; one such example is presented by [24], achieving a sensitivity of ≈−0.0253 μH N^−1^ and a linearity error of ∼5.3%. To enhance magnetic flux linkage, rigid ferrite targets have been utilized; for instance, a ferrite target reported in [42] achieved a force sensitivity of 3.04 μH N^−1^ with a linearity error of 6.5%, though its rigidity limits suitability for conformable integration. Conversely, soft target architectures prioritize flexibility. Magnetorheological elastomers (MREs) introduce field-responsive compliance, albeit requiring specialized magnetic alignment during fabrication [104]. Liquid metal microchannel targets represent a highly compliant alternative that is capable of fluidic deformation and high multi-axis sensitivity; one such study is presented by [74], reaching sensitivities of 2338 μH N^−1^, 2325 μH N^−1^, and 6652 μH N^−1^ along the *x*-, *y*-, and *z*-axes, at the cost of increased encapsulation and patterning complexity.

## 4. Electronic Interfacing Circuits and Readout

Interfacing circuitry is essential for extracting high-fidelity signals from SMI transducers. Because these sensors generate small changes in inductance or oscillation frequency in response to mechanical stimuli, they require dedicated signal conditioning to enhance sensitivity and minimize error propagation. To meet these demands, high-performance readout circuits typically integrate key functions such as low-noise amplification, active filtering, synchronous demodulation, parasitic effect mitigation, protocol conversion, and digital interfacing.

In designing these readout systems, a critical balance must be struck between acquisition speed and signal quality: increasing the sampling frequency accommodates high-speed dynamic tracking, but it introduces higher baseline noise and degrades effective resolution. As validated experimentally in [37], operating an LC oscillator-based inductance-to-digital converter (LDC1614) at moderate sample rates (100 Hz) preserves high resolution, whereas scaling to higher sample rates (4 kHz) facilitates rapid dynamic tracking at the expense of elevated measurement noise. Beyond acquisition trade-offs, environmental stability also impacts measurement fidelity: because the inductive sensor relies on tracking the resonance frequency of an LC tank, temperature variations induce secondary drift by altering the electrical conductivity of both the sensing coil and the target material [106]. This drift complexity is further compounded when scaling from single-element sensors to high-density multimodal tactile arrays, which introduces significant wiring complexity and inter-element crosstalk. To address these scaling challenges, common multiplexing paradigms employ low-resistance CMOS analog multiplexers (e.g., SN74CBTLV3253, Texas Instruments, Dallas, TX, USA) to sequentially switch the I^2^C bus across multiple LDC1614 readout channels [104].

Ultimately, these interfacing trade-offs among sensitivity, power consumption, dynamic range, and electromagnetic compatibility must be wisely balanced to meet the stringent performance requirements of soft robotics and wearable applications. The inductance measurement circuit of an inductive sensor is based on an LC oscillator, the oscillating frequency of which varies with the coil’s inductance [107]:

$$f=\frac{1}{2\pi \sqrt{L\left(C_{\mathrm{ext}}+C_{\mathrm{para}}\right)}}$$

where $C_{\mathrm{ext}}$ is the external tuning capacitance and $C_{\mathrm{para}}$ accounts for parasitic contributions from interconnects and coil winding capacitance. The mechanical perturbation of the coil or the proximity of a conductive or ferromagnetic target modulates *L*, which can be monitored with high resolution as a frequency shift. A widely adopted solution is the LDC1614 inductance-to-digital converter (Texas Instruments, USA) [108], which excites the LC network and digitizes its oscillation frequency [24,33,80,104,109] over a wide operating band (1kHz–10 MHz) with high resolution (28-bit) and low power consumption. The sensing board in [80] uses four LDC1614 devices connected to two I^2^C lines with each chip polling four coils and two chips per I^2^C line. The sensors are interfaced with a Teensy^®^ microcontroller, from which the sensor data are read via an I^2^C bus. Texas Instruments also offers a single-channel, high-speed LDC chip (LDC1101), which can achieve a maximum sampling rate of 180 kS s^−1^ via an SPI communication bus. In [110], Narayanan et al. presented a novel inductance-to-digital converter (LDC) that outputs a digital value corresponding to the change in inductance (ΔL), which is independent of offset inductance, offset mismatch, and series coil resistance. The converter demonstrates good repeatability with a resolution of 7.8 μH for ΔL and 39.1 mΩ for $R_{\mathrm{Ci}}$. To exclude the need for an external driver stage and/or ADCs, Asif et al. [111] present a system that directly interfaces inductive sensors with resolution in the microhenry range.

Beyond wired solutions, wireless readout [38] has emerged as a compelling strategy for a wireless, battery-less readout of inductive modules [66,86,96,112,113], particularly in wearable or biomedical sensing contexts (as addressed in Section Wireless and NFC-Based Sensors). This way, the sensing coil simultaneously serves as the near-field communication (NFC) antenna; variations in its inductive properties modulate the near-field impedance and resonance characteristics, which can be demodulated by an external NFC reader through inductive coupling at 13.56 MHz or similar bands [114]. The integration of NFC enables seamless interaction with portable devices such as smartphones, significantly broadening the accessibility of inductive mechanical sensing technologies in teleoperation, human–machine interfaces, and health monitoring. A comprehensive comparison of key performance metrics across reported readout schemes is summarized in Table 4.

Additionally, NFC-based readout can harvest radio frequency (RF) energy to power minimal electronics or passive modulation circuits. This method not only eliminates tethered connections but also significantly reduces system footprint, giving an advantage for soft, conformal sensors embedded in textiles, wearable skins, or implantable devices.

**Table 4:** Comparison of key performance parameters of various readout schemes.

Readout Scheme	Resolution	Max Sampling Rate	Power/Supply Voltage	Interface Type	Ref.
LDC1614	28-bit	4.08 kSPS	2.7 V to 3.6 V	$I^{2}C$ (up to 400 kbit s^−1^)	[80]
LDC1101	16- or 24-bit	180 kSPS	1.8 V to 3.3 V	SPI (up to 16 MHz)	[115]
LDC1314	12-bit	13.3 kSPS	2.7 V to 3.6 V	$I^{2}C$ (up to 400 kbit s^−1^)	[116]
Enhanced Inductance-to-Digital Converter	7.8 μH	∼11.55 Hz	Low-power (digital processor limited)	Direct sensor-to-microcontroller interface	[110]
Wireless Transfer	3.83 μH (Max)	13.56 MHz	5V, 1A	Hands-free NFC interface	[112]

## 5. Design and Fabrication Methods for SMI Sensors

The performance, versatility, and applicability of SMI sensors are strongly influenced by the design techniques employed. Manufacturing processes not only determine key sensor characteristics, such as flexibility, stretchability, sensitivity, and durability, but also directly affect the production costs, scalability, and system-level integration of sensor arrays into wearable and robotic platforms [117]. The principal design strategies reported in the literature include PCB fabrication, soft lithography, CMOS technology, and textile-based techniques, each offering distinct advantages and limitations. Furthermore, the selected fabrication method is closely linked to the class of conductive materials that can be utilized for coil formation. Highly conductive materials such as copper and liquid metals are commonly preferred to achieve low electrical resistance and high inductive performance, whereas conductive composite materials are generally less suitable due to their significantly lower electrical conductivity.

### 5.1. Printed Circuit Board

**Figure 4:**
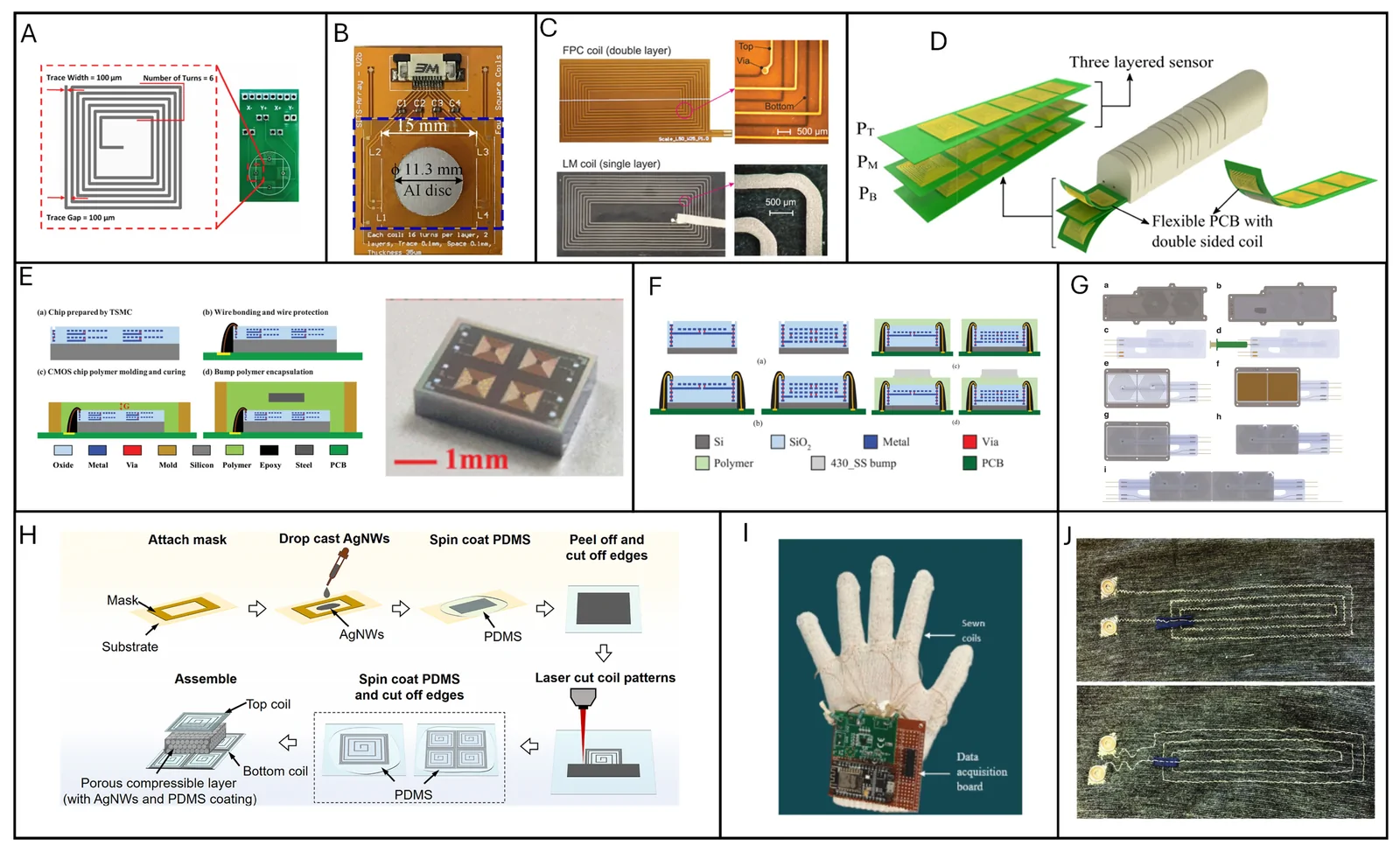
Fabrication techniques of SMI sensors: (**A**) Coils fabricated on a rigid FR4 substrate. Reproduced with permission from Ref. [67]. Copyright (2022) IEEE. (**B**) Two-layer flexible coil on a compliant substrate for a three-axis inductive sensor. Reproduced with permission from Ref. [76]. Copyright (2020) IEEE. (**C**) Images of the fabricated flexible printed coil and liquid metal coil. Reproduced with permission from Ref. [37]. Copyright (2020) Wiley. (**D**) Thin, three-layer flexible sensor with coils etched on both sides and integrated into a soft robotic finger. Reproduced with permission from Ref. [81]. Copyright (2024) IEEE. (**E**) CMOS-based inductive tactile sensor fabricated using the standard TSMC 0.35 μm 2P4M process with the fabricated sensor shown on the right. Reproduced with permission from Ref. [109]. Copyright (2019) IEEE. (**F**) CMOS-based fabrication using the standard 0.18 μm 1P6M process. (**a**) Chip out from Taiwan Semiconductor Manufacturing Company (TSMC). (**b**) Wire bonding and protection. (**c**) Polymer molding and curing. (**d**) Bump assembling and demolding. Reproduced with permission from Ref. [88]. Copyright (2025) IEEE. (**G**) Fabrication of liquid-metal coils using a 3D-printed mold. (**a**) mold, (**b**) mold with silicone, (**c**) rigid ends, (**d**) liquid metal filling process, (**e**) filled sample, (**f**) granular core (GC), (**g**) sample assembly, (**h**) half sample with pneumatic ends, (**i**) ready sample. Reproduced with permission from Ref. [138]. Copyright (2024) SAGE Publications. (**H**) Schematic illustrations of the fabrication process of dual tri-axial tactile sensors. Reproduced with permission from Ref. [30]. Copyright (2025) OAE Publishing Inc. (**I**) GloveSense hand-gesture recognition system with conductive threads sewn onto a glove. Reproduced with permission from Ref. [147]. Copyright (2023) IEEE. (**J**) An inductive sensor with the zigzag and straight stitches. Reproduced with permission from Ref. [151]. Copyright (2021) IEEE.

Printed circuit board (PCB) coils are made directly onto dielectric substrates using conductor traces. The trace dimensions and layout pattern determine the total inductance. They can be fabricated in a single layer [24,118,119], which is the most common approach, or multiple layers [80], to achieve higher inductance. PCB coils offer a compact design and high inductance properties at low production costs. PCBs can be fabricated by commercial prototyping services, as shown in Figure 4A [67], or in-house via laser cutting, as shown in Figure 4B [76], which allows the precise ablation or sintering of conductive traces on both rigid and flexible substrates. They offer high resolution without requiring complex molds or masks.

The most common substrate used to fabricate rigid coils is flame retardant 4 (FR4) PCB. Dacre et al. [80] presented a PCB hosting 16 coils, each with a coil diameter of 25 × 25 mm. The four-layer coils are printed on a rigid FR4 substrate with the track width of 0.1 mm. The coils exhibit consistent behavior, very low crosstalk, high repeatability, and on average, hysteresis below 15%. Despite their ease of fabrication and low costs, the main limitations of these inductive sensors are rigidity, which introduces a mechanical mismatch when interfacing with soft or deformable surfaces.

To overcome these issues, flexible PCBs have been explored [24,33,120,121]. Flexible coils are produced either by using a thin FR4 substrate [81,122,123] or Kapton substrates [76,124]. Wang et al. [37] proposed flexible printed circuit (FPC) coils for folding and bending detection, as shown in Figure 4C. As a comparison, they also made liquid metal coils by direct printing, and the results show that the two types of coils have almost the same inductance change to folding angle. Figure 4D presents a flexible robotic finger developed by Kar et al., in which a thin flame retardant 4 (FR4) substrate was used to fabricate a double-sided flexible PCB [81]. Wang et al. [76] reported four-square double-layer coils which were fabricated on a 25 μm thick Kapton polyimide (PI) film using a standard flexible PCB manufacturing process, which is located in an area of 15 × 15 mm and with a total thickness of 0.23 mm, trace width and pitch of the coils as 0.1 mm. In addition to flexibility, the sensor design can also be adapted for limited stretchability. For example, ref. [123] introduced a novel V-shaped inductive strain sensor with a stretchability of 300% and a low hysteresis of 0.3%. A 6 mm × 6 mm coil was fabricated on an FPC. The crease regions use a 0.12 mm PI film, while the facet regions employ a 0.35 mm reinforced PI film, creating a stiffness gradient that localizes deformation at the creases. The reinforced PI facets measure 7 mm × 7 mm.

While PCB fabrication offers a cost-effective alternative for rapid prototyping with a good balance of feature resolution and fabrication speed, the applications of these sensors on non-planar surfaces are limited due to their lack of stretchability and miniaturization.

### 5.2. CMOS Technology

Integrating inductive sensing with CMOS technology [88,109,125,126,127,128] provides a pathway for developing miniaturized, low-power, and cost-effective tactile systems. CMOS integration enables on-chip signal processing, multimodal sensing, and wireless communication, making inductive sensors practical for large-scale deployment in wearable devices, implantable systems, and soft robotics. CMOS-based sensors combine high sensitivity and stability with compatibility for existing electronic platforms.

CMOS is characterized by predefined processes that affect the circuit speed and power, while the number of metal layers affects the coil QF and sensing performance. A higher number of metal layers offers better routing density, while a lower number of layers is suitable for simpler, cost-effective designs. Typically for inductive sensing applications, Taiwan Semiconductor Manufacturing Company (TSMC)’s 0.35 μm 2P4M [109,127,129], and TSMC 0.18 μm 1P6M [88,128,130,131] CMOS processes are used. Yeh et al. [109] developed a micro three-axis inductive tactile sensor combining a TSMC’s 0.35 μm 2P4M CMOS chip, 2 × 2 coil array, and stainless steel sheet with polymer encapsulation. The fabrication steps can be seen in Figure 4E. With a large sensing range, the sensor distinguishes multi-directional tactile loads with sensitivities of 2.9 nH N^−1^ (normal, *Z*-axis), 17.4 nH N^−1^ (shear, *X*-axis), and 15.3 nH N^−1^ (shear, *Y*-axis). To investigate multiple metal layers in the TSMC 0.18 μm 1P6M CMOS platform, ref. [88] introduced a multilayer interdigitated spiral structure that supports inductive force and capacitive proximity sensing on a single CMOS chip (Figure 4F). In force mode, a single spiral coil achieves 5.1 nH N^−1^ sensitivity, and eight coils enhance sensitivity up to 64×. In proximity mode, interdigitated electrodes detect displacements with a sensitivity of 0.54 fF mm^−1^. Implemented with TSMC 0.18 μm CMOS and post-processing, this design offers a compact, multifunctional solution for tactile applications.

Overall, CMOS integration not only miniaturizes inductive sensors but also enables multifunctionality, high sensitivity, and scalable manufacturing, paving the way for advanced wearable electronics, robotic skins, and implantable haptic systems. However, they are typically fabricated on rigid silicon substrates and therefore lack intrinsic flexibility and stretchability. This mechanical mismatch limits their direct applicability in soft, deformable, and wearable systems.

### 5.3. Soft Lithography

To overcome fabrication limitations in stretchable and flexible inductive sensors, recent advances have focused on techniques such as soft lithography. Soft lithography encompasses various fabrication approaches (e.g., printing, molding, and inkjet) to fabricate microstructures and patterns using an elastomeric mold or substrate [132]. Therefore, it enables the direct integration of conductive coils within soft materials of inductive sensors, allowing them to undergo large mechanical deformation without any loss of electrical functionality. Most of these systems include a silicone matrix and a conductive phase, most commonly liquid metals, while more temperature-independent and stable options include silver nanowires (AgNW) [30] and conductive steel fibers [112].

Common soft lithography approaches include the following: (i) creating silicone molds via 3D printing, such as hollow coils and microchannels, and filling them with conductive materials [66,91,133,134,135,136,137]—one example of the design with the mold process can be seen in Figure 4G [138]; (ii) depositing conductive materials on high-wettability polymer substrates via inkjet and screen printing [139], and (iii) direct printing [140,141] of the coils. This last technique enables the fabrication of much thinner liquid–metal traces, significantly increasing coil density. For instance, ref. [140] used a direct printing method to pattern a liquid-metal coil on a silicone elastomer. The coil was designed with a width of 350 μm, a pitch of 480 μm, and a diameter of 24 mm with each coil layer comprising 21 turns. The overall soft coil structure, consisting of two coil layers, had a diameter of 32 mm and a thickness of 3.5 mm. The main limitation of this design is that even with multiple printing passes, the achievable coil height remains limited, thereby degrading the electromagnetic performance of flexible inductive coils.

Chen et al. [30] presented a fabrication flow (as shown in Figure 4H) where an AgNW/ethanol solution is drop-casted through a shadow mask onto glass, partially embedded into a spin-coated PDMS matrix (∼10 μm depth) for electromechanical stability, flipped for high-precision laser coil patterning, and subsequently encapsulated in PDMS while sandwiching a porous compressible layer (c-PCL) to assemble soft tri-axial tactile sensors. Notably, despite the specific technique adopted, the design freedom offered by soft lithography enables the realization of new functionalities. For instance, Li et al. [142] proposed an anisotropic inductive liquid-metal sensor (AI-LMS), which was fabricated as high-density 3D curved coils using 3D-printed molds to form PDMS and Ecoflex units with microchannels filled with liquid metal. By mimicking fingertip tactile structures through slanted micro-lamellae, the design achieves spatial anisotropy where directional mechanical deformation alters the coil’s inductance. Consequently, AI-LMS showed opposite signal responses under positive and lateral pressures. Both Ecoflex- and PDMS-based AI-LMS sensors exhibit excellent linearity over low pressure ranges up to 0.756 kPa (0.107 N) and 7.055 kPa (1 N), respectively, with high $R^{2}$ values of 0.999 and 0.997.

Recent efforts have focused on extending the advantages of soft lithography and liquid metals in multilayer coils. Ref. [39] proposed an induction-based sensor array employing liquid-metal ink coils printed on an elastomer substrate. The distance between adjacent liquid-metal lines was 500 μm. The bottom coil exhibited a width and height of (352.0 ± 17.5) μm and (43.3 ± 3.1) μm, respectively, while the top coil measured (218.0 ± 14.6) μm in width and (86.2 ± 2.8) μm in height. This design enables high deformability and conformal integration on 3D surfaces. To increase the number of layers, Su et al. [143] presented a multilayer high-density liquid-metal coil (MHD-LMC) in conjunction with a flexible, electrically insulating PDMS substrate, featuring a channel height of approximately 230 μm beneath a 450 μm-thick PDMS film. Through optimized patterning design and precise interlayer alignment, MHD-LMCs with up to 20 layers were successfully fabricated.

### 5.4. Textile-Based

Textile-based fabrication offers an alternative approach for fabricating SMI sensors by embedding conductive fibers or yarns directly into textile substrates. Conductive fibers, such as metal-coated yarns or carbon nanotube-based threads, are incorporated during the textile manufacturing process to form inductive pathways within the fabric.

A key advantage of textile-based inductive sensors is their suitability for long-term everyday use, particularly in medical and wearable applications [144,145]. Unlike surface-mounted sensors, conductive threads can be sewn [68,146,147,148] or knitted [93,94,149,150] directly into garments, allowing sensors to conform naturally to the body and maintain functionality under repeated deformation. In a wearable device called GloveSense [147], as shown in Figure 4I, pairs of copper coils are sewn on both sides of the fingers of a glove and connected in series. It used conductive threads (diameter of 0.13 mm) for the first time to produce coils with high accuracy and fast response time for hand gesture recognition. They have demonstrated three configurations of the coils: a helical coil wound around the finger, a rectangular coil placed along the finger, and two rectangular coils on each finger. Knitted coils have emerged as innovative breathing sensors, leveraging their unique inductive properties to monitor respiratory patterns effectively. One representative example is presented by Mouckova et al. [151], who fabricated an inductive sensor on a knitted fabric using an industrial embroidery machine. As illustrated in Figure 4J, the sensor utilizes a hybrid conductive yarn containing eight silver-plated copper microwires. Fobelets et al. proposed knitted coils that integrate insulated Litz wire with elastic yarn, allowing flexibility and stretchability, which is crucial for accurate breathing monitoring [150].

Existing single-layer textile inductive sensors suffer from low sensitivity, limited force range, difficult layer alignment, and coil fracture caused by property mismatches between soft fabrics and conductive materials during wear or washing. To improve durability, recent studies focus on encapsulation, yarn-level protection, and elastic conductors. Blanket encapsulation using PDMS coatings shields conductive fabrics against washing abrasion [152]. At the yarn level, embedding electronics directly into stainless steel filaments and protecting solder joints with heat-shrinkable tubing maintains integrity through laundering cycles [153]. During manufacturing, screen-printing highly elastic conductive inks allows traces to stretch with fabric fibers, reducing wash fatigue [154]. Similarly, chemically modifying conductive polymers (e.g., PEDOT:PSS or polyaniline) via doping or grafting enhances trace elasticity, preventing micro-cracking and wash fatigue without sacrificing wearability [155].

A key route to improve the performance, reliability, and cost-effectiveness of SMI sensors lies in the simplification of their fabrication workflows. In particular, minimizing fabrication complexity by reducing the number of processing and assembly steps, or adopting fully integrated manufacturing strategies, can significantly enhance their suitability for sensor-integrated soft robotic systems and wearable technologies. As summarized in Table 5, the choice of fabrication technique is strongly associated with key design parameters, including coil track width, material selection for both coils and substrates, and achievable sensing area scaling.

**Table 5:** Comparison of different design techniques and their scaling.

Design Technique	Track Width	Substrate Material	Coil Material	Sensing Area	Ref.
PCB design	35 μm	FR4	Copper	D=15 mm	[67]
	0.1 m m	FR4	Copper	625 mm^2^	[80]
	35 μm	Flexible FR4	Copper	102 mm^2^	[81]
	254 μm	FR4	Copper	Coil length = 76 mm	[122]
FPC	100 μm	Kapton (PI)	Copper	225 mm^2^	[76]
	300 μm	PI	Copper	36 mm^2^	[123]
	130 μm	Fabric	Insulated copper thread	1200 mm^2^	[147]
3D printing	300 μm	Silicone	Liquid metal	200 mm^2^	[156]
	*D* = 680 μm, 1200 μm and 1700 μm	PDMS	Liquid metal	Coil length = 20 mm	[157]
	*D* = 672 μm and 679 μm	PDMS/Ecoflex	Liquid metal	142 mm^2^	[142]
	350 μm	Ecoflex	Liquid metal	1250 mm^2^	[66]
Soft lithography	350 μm	Silicone	Liquid metal	D=12mm	[140]
	350 μm	Silicone	Liquid metal	D=24mm	[43]
	352 μm	PDMS/Ecoflex	Liquid metal	D=20mm	[39]
	230 μm	PDMS	Liquid metal	$V=132.35mm^{3}$	[143]
CMOS	7 μm	Silicon	Copper	5.6 mm^2^	[109]

## 6. Sensing Modalities

SMI sensors have attracted significant attention due to their unique combination of mechanical robustness, high sensitivity, and environmental stability, making them suitable for a broad spectrum of applications. The fundamental working principles described in Section 2 enable a variety of sensing modalities. The following subsections discuss how these principles are leveraged to measure distinct physical phenomena, which is supported by representative examples. Moreover, a short section is dedicated to temperature sensing. Finally, Table 6 summarizes these modalities, highlighting state-of-the-art works that demonstrate superior performance in terms of resolution, sensitivity, and dynamic range. Because these devices are evaluated under diverse testing conditions, Table 7 provides a detailed parameter-by-parameter comparative analysis, including spatial dimensions, excitation frequency, readout electronics, noise floor, hysteresis, and response time, offering readers a clearer understanding of the hardware trade-offs across implementations.

### 6.1. Mechanical Sensing

#### 6.1.1. Strain and Proximity

One of the most direct sensing modalities derived from the inductive sensing principle is strain and proximity sensing. The electromagnetic field generated by the coil is affected by the presence of the target in its vicinity, which defines a direct relationship between the target distance and the measured inductance. When the interaction occurs at the sensor surface, the resulting inductance variation is associated with strain; otherwise, it corresponds to the proximity of the same metal target.

This modality is useful in different cases, including proprioception in robotic manipulators [25,26,158], monitoring in prosthetics and rehabilitation [159], precision medical robotic tasks like needle insertion [160], and feedback in electromagnetic actuators like voice coils and autofocus lenses [161].

Researchers in [161] achieved the best resolution at 0.038 μm and the highest sensitivity at 3.9 nH μm^−1^, whereas the sensors proposed in [25,26] are based on a variable-spring inductance whose sensitivity depends on the elongation state. In terms of sensing range, the researchers in [159] achieve the targeted displacement range of 0 mm to 15 mm appropriate for limb-socket gaps, while the sensor in [26] demonstrates >100% strain range, which is the largest relative range in the state of the art.

Overall, these sensors report low repeatability errors (<0.15%), low noise, and drift. One key aspect that makes these sensors stand out is the minimal hysteresis reported. The maximum value reported by [161] of 1.5% is well below the typical values seen in resistive or capacitive soft sensors. The main design trade-off here is resolution versus operating range. The finest sensors, like the one in [161], operate at μm ranges, while broad-range sensors sacrifice absolute resolution.

#### 6.1.2. Force

Force sensing is essential for applications requiring an accurate detection of tactile interactions. In SMI sensors, force is inferred from mechanical deformation, as an applied load displaces the conductive target relative to the coil, inducing a measurable shift in inductance. SMI sensors are capable of detecting not only normal forces [102,162] but also shear forces [76,82,84,163], enabling more comprehensive tactile perception. One such example is presented by Khalid et al. [67]: a three-axis inductive force sensor that measures normal, shear, and angular forces. Their results show that compared to RTV-528, the Ecoflex-30-based sensor demonstrated an improvement in force resolution of approximately 83.4%, 32.0%, and 72.4% for the normal, shear, and angular shear modes, respectively. Another example is shown in [164], where the soft tactile sensor measures the three force components with adjustable sensitivity and measurement range. Recently, we investigated the possibility of tuning sensitivity and force range harnessing a 3D-printed lattice structure [42]. Our results show that the high-stiffness lattice (20% relative density) offers a larger force range necessary for handling heavier objects, while the softer lattice (7% relative density) provides higher sensitivity required for delicate object manipulation. Chen et al. [30] proposed a three-axis tactile sensor that provides real-time information on both normal and shear forces with high sensitivity (910 nH N−1, 0 N to 1 N) and a wide range (0 N to 12.6 N). The unique hybrid coil design generates a more uniform distribution of magnetic field, which improves the sensor’s lateral shear sensing performance.

#### 6.1.3. Angle

Changes in magnetic field coupling can be produced in space by deforming flexible planar coils. Specifically, by folding or bending a flexible coil alone (i.e., without any target or other deformable material embedded), the respective angle or curvature can be measured. That is how we earlier developed the soft inductive angle sensing (SIAS) [37] transduction method. We were motivated by the limitations of conventional strain-based sensors, particularly their sensitivity to placement and complex mechanical behavior. Our results have shown that SIAS is hysteresis-free, velocity-independent, highly sensitive, and ultrastable, and it also has a fast response, guaranteeing highly precise (0.1° incremental folding angle change) and reliable measurements.

In continuum robots, inductive angle sensors enable 1D [25,51,165,166,167], bidirectional [65,162], and omnidirectional [168] bending sensing. In wearable and biomedical applications, bending sensors are mostly used to monitor joint angles in rehabilitative scenarios [68] and for real-time body-shape reconstruction [26,93,169]. Researchers in [170] provide a comprehensive review of angle measurement sensors beyond soft bending sensors and inductive sensing technologies. The study covers applications in the automotive industry, additive manufacturing, and the design of Computer Numerical Control (CNC) machines.

The sensor in [41] surpasses the state of the art in almost every aspect of angle sensing with a −200° to 327° range at 14.3 nH°^−1^ sensitivity with minimal hysteresis and thermal drift of 0.011°/°C. In terms of sensor resolution, the device in [37] achieves an 11× improvement with a resolution of 0.00087° (15 μrad). The sensor in [93] has the highest raw sensitivity of up to 551 nH°^−1^, but it degrades rapidly outside its optimal operating range.

Overall, soft inductive bending sensors offer robust, contactless sensing capabilities; however, their performance is often constrained by geometric complexity, and sensitivity is affected by the alignment of coil and target, indicating opportunities for further optimization in design and integration.

#### 6.1.4. Shape and Texture Recognition

The accurate perception of object shape and texture is essential for robust manipulation, as these properties directly affect grasp stability, contact quality, and interaction safety in complex and unstructured environments. By providing detailed information about the contact interface, these features enable more reliable object recognition and adaptive manipulation strategies. SMI sensors are a promising solution for these applications due to their compliance and multi-sensing capabilities. Li et al. [167] proposed a soft robotic finger integrating an inductive sensor at the fingertip for the simultaneous perception of shape and texture. The sensing mechanism combined mutual inductance-based bending detection with eddy-current-based force sensing. Owing to its high-resolution tactile and bending sensing capabilities, the system was able to successfully distinguish fine surface features, including small protrusions and ridged patterns with varying geometries and pitches. Similarly, surface topology recognition was also explored by Peng et al. [41] in a magnetic crack-based piezoinductive sensor (MC-PIS) that utilizes changes in magnetic flux caused by strain in cracked ferrite films. The sensor is demonstrated in a soft pneumatic finger by sticking at the bottom surface of the finger actuator, and their results show that by sliding the finger on a surface, the sensor recognizes its topology of sub-mm features (0.6 mm). Furthermore, the system was able to detect localized surface irregularities, such as the last-used position on a Scotch tape roll, through distinct pulse-like inductance signals generated during sliding contact and the sensitivity to vibration to distinguish music notes transmitted from a speaker.

Despite these successful implementations, SMI sensors face limitations regarding spatial resolution and complex texture recognition when compared to other tactile transduction mechanisms. For example, optical tactile sensors can achieve micro-scale spatial resolution, distinguishing surface curvatures as small as 5 m^−1^ and roughness features on the order of 100 μm [171]. In contrast, the spatial resolution limit of SMI sensor arrays is fundamentally bounded by the geometric scaling of the planar coils and inter-coil electromagnetic crosstalk. Minimizing coil footprint to increase taxel (tactile pixel) density drastically reduces the coil self-inductance and QF, severely compromising the signal-to-noise ratio and force sensitivity. Progress in advanced fabrication processes will be key to overcoming these coil scaling limits and enhancing spatial resolution.

### 6.2. Other Modalities

#### 6.2.1. Temperature

Inductive sensing can be exploited to indirectly measure temperature through the changes in electrical or physical properties induced by temperature variation. Such changes, in turn, affect the electromagnetic field generated by the coil, resulting in a measurable change in inductance.

In [157], a linear temperature response was achieved in the range of 27 °C to 100 °C at a resolution of 0.2 °C using the thermal expansion effect of PDMS. On the other hand, the sensor developed in [172] can detect temperatures above 50 °C with a resolution of 0.51 °C by measuring the change in resistivity of a titanium target.

#### 6.2.2. Material Identification

Inspired by human skin, SMI sensors can also distinguish materials based on properties such as thermal conductivity, electrical conductivity [100], or mechanical characteristics. This capability is important for enabling robots to better interpret object identity and improve decision making during manipulation and interaction tasks. One such example, previously mentioned above, is the study proposed by Peng et al. [41], where they distinguish objects’ mechanical properties based on the vibration transmitted through the objects, which is perceived by the artificial finger with the inductive sensors embedded. Li et al. [157] identify the conductive substance as it causes a change in the inductance of the high-density liquid-metal coil (HD-LMC), and it was observed that a positive correlation exists between the magnetic permeability of the metal and the resulting signal variation. Li et al. [172] identify the materials based on the thermal conductivity when the heat transfer occurs between the object and the sensors. Their sensor distinguishes the surface materials among glass, wood, or metals by sensing the temperature changes of the sensor because of different rates of heat dissipation.

A common theme observed is that variations in inductance serve as an effective proxy for quantities that the sensor cannot directly measure. This latent relationship can be exploited further using shallow neural networks to extract additional information from the sensed signal. Such indirect inference mechanisms are central to inductive sensors’ ability to achieve complex perception from a single scalar measurement. However, a key challenge lies in developing robust methods for decomposing and interpreting these signals to enable reliable multimodal sensing.

Recent studies have addressed this bottleneck by pairing flexible coils with multifunctional structural layers, such as piezoresistive MXene/carbon nanotube aerogels, to capture overlapping mechanical, thermal, and magnetic inputs simultaneously [173]. By feeding these coupled multimodal variations into shallow machine learning classifiers, modern frameworks can accurately decouple baseline contact forces from intrinsic material properties, enabling real-world material recognition with accuracies exceeding 99%. Building upon this, next-generation embodied architectures combine compact multimodal sensors, simultaneously capturing pressure, thermal, and dielectric signals, directly with Large Language Models (LLMs) [174]. This integration elevates performance by leveraging semantic reasoning to resolve complex signal ambiguities, achieving an overall recognition accuracy of up to 99.13% across 23 representative materials while translating raw physical features into adaptive robotic control decisions.

**Table 6:** Summary of peak reported performance metrics across different sensing modalities.

Sensing Modality	Highest Reported Resolution	Highest Reported Sensitivity	Widest Reported Range	Lowest Reported Hysteresis	References
Strain and Proximity	0.038 μm [161]	3.9 nH μm^−1^ [161]	0 mm to 15 mm [159]	0.1% [25]	[25,26,158,159,160,161]
Force and Pressure	0.4 mN [162]	39.04 μH N^−1^ [42]	20.07 N [42]	4.7% for shear, 5.8% for normal [76]	[30,42,67,76,82,84,102,162,163,164]
Angle	0.00087° [37]	551 nH°^−1^ [93]	−200° to 327° [41]	0% [41], 0.92% [37]	[25,26,37,40,41,44,51,52,65,68,93,112,162,165,166,167,168,169,170,175]
Texture and Tactility	0.6 mm [41]	—	—	0% [41]	[41,167]
Temperature	0.2 °C [157]	—	27 °C to 100 °C [157]	0.35–0.9s (time-based) [157]	[157,172]
Material Identification	Pressure: 0.357 kPa, Temp: 0.2 °C [157]	—	166 Hz [41]	0.35–0.9s (time-based) [157]	[41,157,172]

Note: These values reflect peak performance metrics reported in individual surveyed studies. These are non-normalized, device-specific values intended to illustrate upper bounds rather than direct comparative performance rankings.

**Table 7:** Comparative analysis of excitation parameters, readout electronics, and key dynamic performance.

References	Excitation Frequency	Dimensions	Readout Electronics	Hysteresis	Response Time	Noise Floor
[161]	0.1 Hz to 100 Hz	D=9 mm, h=2 mm	LDC1614	4.67%	17 ms	0.25 $\mu m_{\mathrm{pp}}$
[162]	100 Hz	58.5 m m	LCR-8210	0%	50 ms	0.28 n H
[42]	0.4 M Hz	25 mm × 25 mm	LDC1614	8.7%	∼81ms	—
[37]	5.3 M Hz	50 mm × 25 mm	LDC1614	<1.0%	—	0.020 nH RMS
[93]	457.6 kHz to 744.5 kHz	—	Wayne Kerr 6500B	—	—	—
[41]	200 kHz	15 mm × 10 mm	LDC1614	0%	6.1 m s	0.05 nH RMS
[157]	100 kHz	L=20 mm	TH2832	0.35 s (time-based)	—	—

## 7. Applications

The flexible coil designs of SMI sensors and their ability to be miniaturized enable easy integration into a wide range of platforms. In the following subsections, we highlight the applications in robotics, wearable and biomedical systems and miscellaneous, illustrating their versatility and potential for next-generation devices.

### 7.1. Wearable and Biomedical

SMI tactile sensors have found applications in wearable and biomedical systems [36,66], where they provide high-resolution, multimodal sensing for real-time monitoring of body movements, forces, subtle deformations [68,76,82,84,112,159,172,176], and prosthetics [177]. Embedding flexible inductive sensors into garments or wearable devices enables the tracking of biological signals [43], gesture recognition [178], rehabilitation monitoring, robotic control via natural motions, surgical tools [67], biomedical implants [86], biochemical sensors [179], and wearable plethysmography systems [93,94,95].

**Figure 5:**
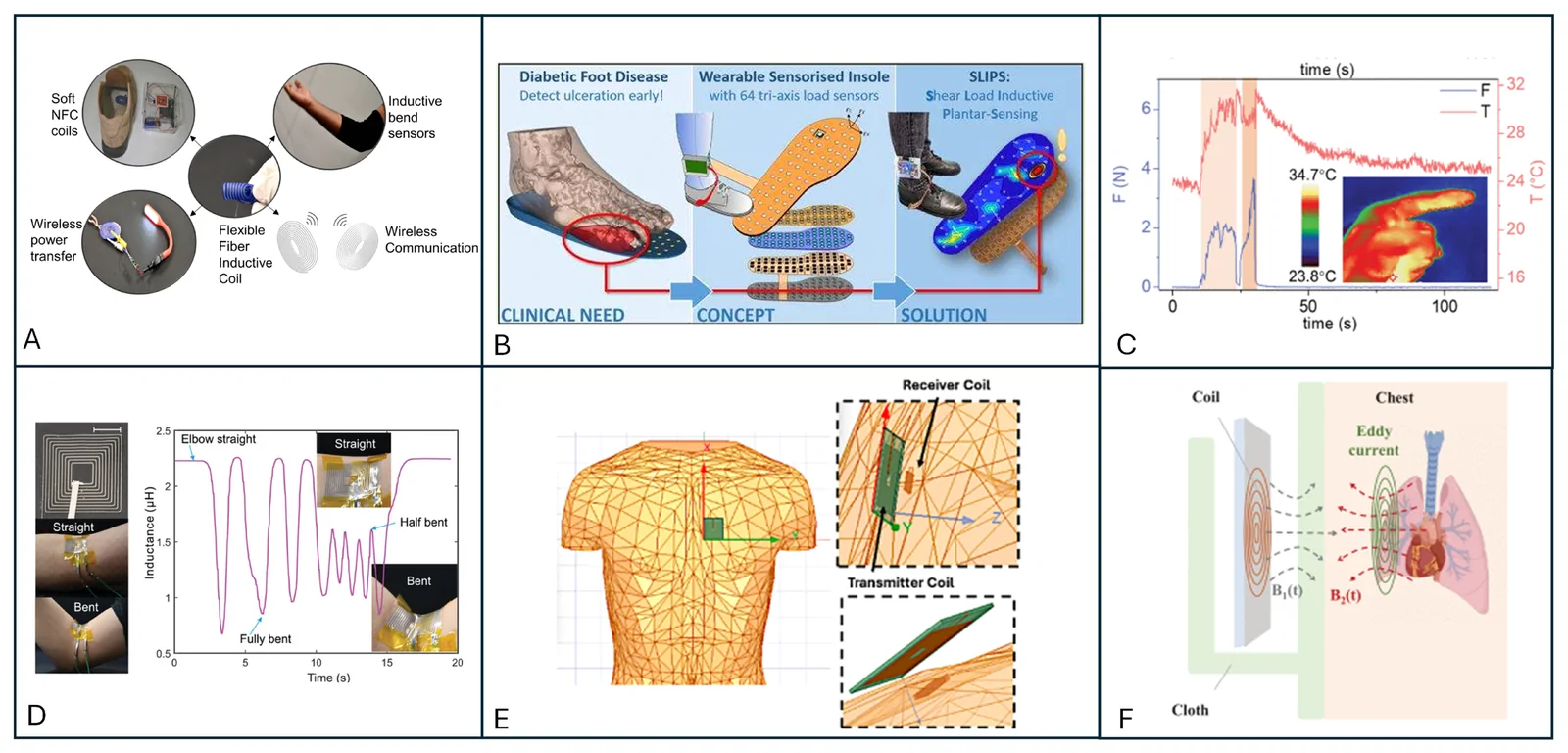
Wearable, biomedical and wireless applications: (**A**) Representative applications of soft inductive coils across different domains. Reproduced with permission from Ref. [112]. Copyright (2022) IEEE. (**B**) The conceptual design of a wearable, sensorized insole with 64 three-axis inductive sensors for the diabetic foot. Reproduced with permission from Ref. [84]. Copyright (2022) IEEE. (**C**) Temperature and force response of an IMTS pressed with a finger with small and strong forces. Reproduced with permission from Ref. [172]. Copyright (2025) IEEE. (**D**) A square liquid-metal coil was attached to the inner side of the elbow to monitor the angle (scale bar: 10 mm). Reproduced with permission from Ref. [37]. Copyright (2020) Wiley. (**E**) The implementation of the multilayer square coils in biomedical implants for WPT. Reproduced with permission from Ref. [86]. Copyright (2025) The Electromagnetics Academy. (**F**) The developed pocket-sized eddy-current-based inductive sensor simultaneously measures heart rate and lung function. Reproduced with permission from Ref. [38]. Copyright (2024) IEEE.

Advances in fabrication techniques have been critical for these applications. Coils developed by Mathai et al. [112] shown in Figure 5A endure large elastic deformations (minimum bending radius ≤ 1 mm) and are versatile for applications in flexible sensors, wireless charging, soft NFC tags, and wearable electronics. This approach supports robust soft robotic and human–machine interface designs, offering a flexible alternative to rigid PCBs. Building on these advances, several works demonstrated inductive sensing for biomechanical applications [76,82,84], specifically plantar load monitoring. To capture full-foot loading in real-world scenarios, Wang et al. [84] developed the SLIPS system as pictured in Figure 5B, integrating 64 three-axis sensors into a flexible insole. Pilot experiments revealed that shear and pressure distributions are complex and often non-overlapping, emphasizing the importance of simultaneous multiple axes measurement for clinical assessment and preventive care.

Li et al. [172] further expanded the capabilities of inductive tactile sensors by introducing an inductive multimodal tactile sensor (IMTS) that simultaneously measures force and temperature through eddy-current coupling. With high resolution (1.46 mN for force, 0.51 °C for temperature) and fast response, the IMTS can detect subsurface material properties, wetness, and airflow when integrated into soft robotic fingers, demonstrating potential for robotic tactile sensing and intelligent biomedical devices (Figure 5C).

Wang et al. [66] addressed the challenge of integrating inductive sensing into stretchable and wearable platforms by demonstrating that planar coil AR is critical for tuning strain sensitivity. By designing strain-invariant and high-AR coils, the sensors maintain linear, hysteresis-free responses under large deformations and varying environmental conditions. This enables the precise monitoring of fine human movements, muscle fatigue, grasping force, and hand gestures, making them ideal for soft robotics, wearable health monitoring, and real-time biomechanical tracking. Moreover, Wang et al. [37] attached a liquid-metal coil to the inner side of the elbow (as shown in Figure 5D) to measure joint angle, enabling applications in body-motion and gesture monitoring.

Recent designs utilize breathable, gas-permeable substrates like electrospun fibrous networks or porous silicones to prevent sweat accumulation and skin irritation [180]. Taking wearability further, Nie et al. [181] demonstrated a textile-based wireless pressure sensor array (WiPSA) built on flexible 3D fabric spacers with excellent environmental stability; its resonant frequency shifts by only 2.4% under extreme temperatures (15 to 103 °C) and 2.6% under high humidity up to 99%. Finally, to address long-term signal drift, researchers are focusing on evaluating different circuit topologies and structural designs under stable temperatures. The study by Lu et al. [182] introduces an innovative, ADC-less digital approach by comparing a custom discrete resonance circuit against an integrated inductance-to-digital converter (LDC) chip, discovering that the resonance circuit architecture yields superior long-term time drift stability. By combining this method with a structural magnetic shield ring to optimize the probe’s sensitivity and QF, the framework successfully achieves an ultra-low, highly stable time drift root mean square (RMS) of 0.01 μm per 24 h under controlled temperature conditions.

Although these wearable and robotic systems demonstrate the flexibility, multimodal sensing, and high precision of inductive sensors, their translation to large-scale deployment or implantable devices requires compact, low-power, and scalable electronics. This motivates ongoing research into CMOS-based inductive sensing platforms that can integrate seamlessly into next-generation wearable and biomedical applications.

#### Wirelessand NFC-Based Sensors

Compared to other components of mechanical sensors, coils offer a versatile solution for improving sensor electronics, including wireless charging units, soft NFC tags, and communication devices. The study by Mathai et al. [112] shown in Figure 5A aids sensor communication through the use of a flexible fiber inductive coil for NFC and short-range communication applications. In medical applications [183], Hsu et al. [38] present a pocket-sized non-contact sensor for cardiac and lung monitoring. By placing the sensing coil with an alternating magnetic field B1(t) on the chest, the coil induces eddy currents in the biomedical target, validating the ability to measure both cardiac and lung signals without direct contact with the skin. Figure 5F shows the schematic of their proposed sensor where the sensor is placed on the chest to record both the cardiac and lung signal. The sensor was compared with the commercially available chest-worn respiratory sensor. To facilitate wireless electronics, a study by Abduljaleel et al. [86], as shown in Figure 5E, presents a power transmission system for biomedical implants by reducing the receiver coil size (10 mm) while maintaining high efficiency. The system demonstration shows an impressive transfer efficiency of 87.9%. Since 2D coil design limits the use of wireless transfer, Hou et al. [96] fabricated a 3D coil for WPT.

These applications can motivate simpler and lighter designs, which improve the scalability of inductive sensor arrays and enable larger, more complex systems with reduced power consumption. They also support the development of more effective human–machine interfaces, fostering better collaboration between humans and machines in industrial and medical environments.

### 7.2. Robotic Gripping and Manipulation

**Figure 6:**
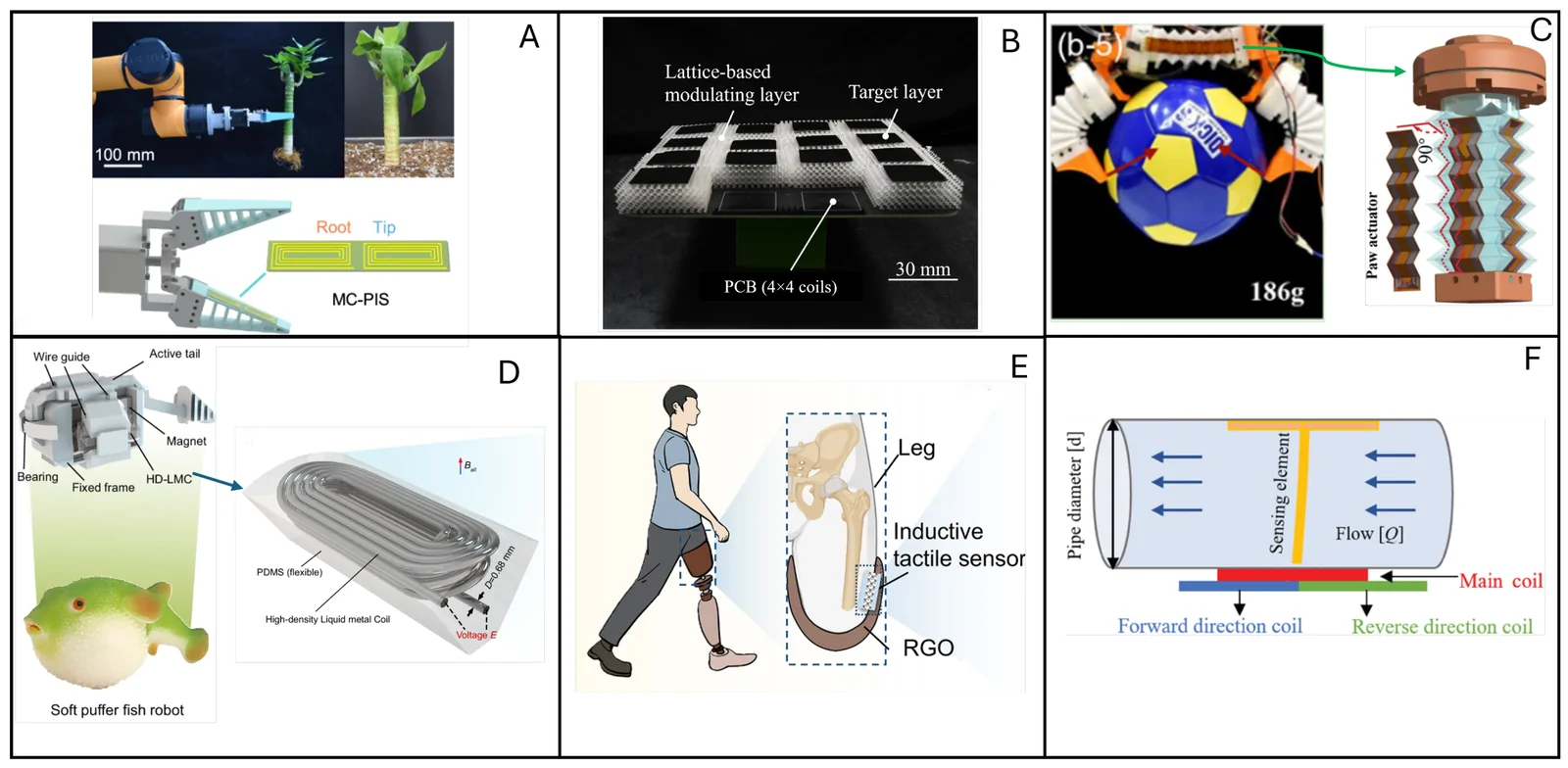
Applications of SMI sensors in robotic grasping, manipulation, HMI, and miscellaneous systems: (**A**) Soft fin-ray gripper with two integrated MC-PISs at the tip and root of the finger. Reproduced with permission from Ref. [41]. Copyright (2025) Nature. (**B**) Photograph of the COPESS-based surface manipulation setup, consisting of a sensing unit with a 4 × 4 coil array, a lattice-based deformable layer, and a target layer. Reproduced with permission from Ref. [42]. Copyright (2026) IEEE. (**C**) Intelligent soft gripper enabled by the origami module with the compliant paw actuator. (b-5) represents the paw is pressurized for grasping the largest size and weight. Reproduced with permission from Ref. [40]. Copyright (2026) Wiley. (**D**) Structural design of the flexible bionic pufferfish with integrated HD-LMC. Reproduced with permission from Ref. [157]. Copyright (2024) Nature. (**E**) Three-axis tactile sensors in monitoring the normal and pressure loads between the leg and RGO. Reproduced with permission from Ref. [30]. Copyright (2025) OAE Publishing Inc. (**F**) The conceptualized design of the inductive sensing-based flow measurement sensor with three stationary planar coils attached externally to the pipe. Reproduced with permission from Ref. [44]. Copyright (2024) IEEE.

In the field of robotic manipulation, SMI sensors have proven useful in different main use cases. The first is proprioceptive shape sensing. This is demonstrated in [26] where the sensors are embedded as structural fiber replacements in a pneumatic arm, in [25], to close the control loop of a tendon-driven flexible manipulator, and in [40], to build a modular gripper around foldable inductance feedback. The second use case is tactile sensing, as demonstrated in [51,167] and [42] for extracting the object properties and contact force during manipulation tasks, in [168] for drawing geometric shapes and writing characters, and in [162] for typing tasks. The third use case is contact-alignment sensing for improved end-effector control. Researchers in [175] target the pre-grasp phase of the end effector, detecting tilt before full contact is established. A tree planting gripper is developed in [41] that performs the full sequential manipulation task to plant a tree (Figure 6A). Figure 6B shows a lattice-based soft surface with the sensors embedded to manipulate the objects on the top and obtain the localized position of the object.

Another interesting aspect is the structural integration demonstrated in the work in [26], where the sensors replace the pneumatic fiber reinforcement, as well as in the study in [25], in which the spring is routed through existing channels, and in [51], where the PCBs are mounted onto rigid fins already embedded within the soft finger. In these approaches, the sensing elements are not merely externally attached to the actuator but are incorporated into the load-bearing structure itself, representing a significant architectural advantage over conventional resistive or optical sensing alternatives.

Despite their advantages, SMI sensors integrated in robotic manipulators exhibit several limitations. Angle sensitivity decreases in near-flat configurations, while single-axis ambiguity remains a persistent challenge for accurate pose estimation [25,51]. Some studies, therefore, rely on vision-based ground truth for sensor validation. Furthermore, the neural-network inference approach in [40,168] shows reduced accuracy near zero pressure due to sparse training data. These limitations could be mitigated through the integration of multiple sensors, recurrent neural networks, or physics-informed learning approaches.

Mechanical matching between sensors and actuators lies at the core of soft robotics design. To preserve the actuator’s baseline performance, the integrated sensor must remain sufficiently compliant (i.e., possess a low Young’s modulus) so as not to increase the overall structural stiffness or degrade net output force. However, sensing elements are frequently added post-fabrication, creating interfacial mechanical mismatches that compromise compliance and induce structural failure under cyclic loading. To overcome this limitation, advanced fabrication methods and material formulations must enable sensing elements to be monolithically integrated into the soft body. Ideally, the sensing mechanisms should originate directly from the robot’s architectural design or be realized using the same elastomeric materials as the host soft body [184].

### 7.3. Bioinspired Locomotion

Researchers have long sought to understand nature for more energy-efficient locomotion modes, especially in diverse environments such as underwater settings. With growing interest in soft robotics, bioinspired locomotion designs are gaining popularity. Embedding sensors is fundamental because they provide information to correct the bioinspired motion when the system encounters obstacles or changing conditions. The examples below illustrate how inductive sensing fills this role across different locomotion strategies. For instance, a puffer fish-inspired aquatic robot achieves undulatory and rolling motion along a magnetic track in [157]; here, the liquid-metal solenoid that drives the wheel via Lorentz force doubles as a rotary encoder, allowing the system to achieve closed-loop control directly from its own actuation element. In another work, a buoyancy-driven jellyfish-inspired robot, which is also demonstrated as an intelligent gripper (Figure 6C), relies on length- and angle-sensing modalities to achieve proprioception of its folding motion [40]. Researchers use peristaltic crawling motion in [41,65] and incorporate an inductive sensing mechanism in their designs to support a richer set of behaviors: the crawling robots further exhibit a set of behavioral reflexes, such as playing dead [41,65], escape, and obstacle crossing (only in [41]), through the tactility and force-sensing modalities. In [65], simultaneous proprioception and force sensing are achieved through frequency multiplexing. Despite achieving some level of biomimicry, these works still have a long way to go toward their goal. Closed-loop control is achieved only in [157], whereas the others rely on reactive sensing.

### 7.4. Human–Machine Interface

Human–machine interfaces (HMIs) play a critical role in enabling intuitive communication and interaction between humans and robotic systems. To achieve seamless and comfortable interaction, sensing technologies must be flexible, lightweight, and capable of accurately detecting complex human motions and touch-based inputs [185]. Owing to their inherent compliance, deformability, and high sensitivity to mechanical stimuli, SMI sensors have emerged as promising candidates for next-generation interactive HMI systems [65,112,164]. The work proposed by Chen et al. [30] is one such example, as shown in Figure 6E: a three-axis tactile sensor provides real-time information on both normal and shear forces and is demonstrated between the user’s leg and the orthosis, showing excellent stability and resistance to environmental contaminants. Many devices mentioned in the wearable and biomedical application section can be demonstrated in human–machine interaction. For example, Byberi et al. [147] proposed a wearable sensing device, named GloveSense, in which insulated copper threads were sewn into a glove to form inductive coils capable of tracking finger movements. The system demonstrates strong potential for integration into prosthetic and human–computer interaction (HCI) applications by enabling intuitive and wearable motion sensing. In the future, it will be interesting to explore how such SMI sensing systems can be further utilized for immersive interaction, gesture-based control, and real-time haptic interfaces.

### 7.5. Miscellaneous

Beyond their significant applications in wearable electronics, biomedical systems, and robotics, SMI sensors have also demonstrated strong potential in a wide range of miscellaneous applications. One such example is the sensor proposed by Zhang et al. [83], where a flexible wide-range multidimensional force sensor is demonstrated in a Morse Code system for directional awareness. Peng et al. [41] demonstrated their piezo-inductive sensor to distinguish the musical notes as the sound waves traveled through the desk. Similarly, Jin et al. [43] proposed a liquid metal-based stretchable coil that worked simultaneously as a loudspeaker and a microphone. Another example is the system shown in Figure 6F, which measures the water flow rate in a pipe using a cantilever structure that deflects under fluid flow. Flexible coils are employed to sense the resulting bending angle [44]. The sensing system consists of three flexible PCBs, with a central coil and two auxiliary coils (forward and reverse) positioned on either side, each overlapping 65% with the main coil. Over a deflection range of 0° to 34°, the system is capable of measuring flow rates from 0 to 7.1 m^3^ h^−1^ with an accuracy of 3%.

Although the previous section presented a broad range of sensing modalities enabled by inductive sensors, only a limited subset of these modalities is commonly utilized in practical applications. To fully utilize SMI sensing, improved signal decomposition methods are needed to extract different types of information from a single sensor signal. Furthermore, the application of learning-based approaches remains relatively limited in the current state of the art despite their significant potential to enhance performance in complex tasks and to transition toward large-scale multimodal sensor arrays.

## 8. Conclusions: Challenges, Opportunities and Future Direction

One of the main challenges of SMI sensors is their susceptibility to EMI. Since these sensors operate in time-varying magnetic fields, external conductive devices, such as metal objects, and electromagnetic sources, such as power lines, motors, and wireless communication devices, can induce parasitic signals in the sensing coils. EMI and environmental noise can distort measured inductance, particularly in high-sensitivity regimes. To mitigate these effects, Decre et al. [80] employed a 0.1 mm thin ferrite sheet beneath sensing coils to shield against motor-induced EMI. Alternatively, conductive polymer composites [186] and advanced nanomaterials, such as MXene/silver nanowire networks on silk textiles [187], provide high-performance EMI shielding. Noise can also be cancelled architecturally: differential measurement structures with symmetric dual coils reject common-mode noise and thermal drift, achieving up to a nine-fold sensitivity gain [188]. Additionally, active-drive phase architectures eliminate injection pulling to enhance system-level EMI robustness [189].

Further, coils for inductive sensors are made of conductive materials such as copper, aluminum, or liquid metals. Because of the temperature increase, the electrical resistance increases, leading to thermal expansion of the coil, changing the net inductance [123].

Another major limitation of the inductive sensors is their relatively high power consumption compared to the passive sensing approaches. Continuous high-frequency excitation and inductance readout circuits increase energy demand, particularly in high-density arrays. This poses challenges for soft robotics and wearable systems with limited battery capacity. Additionally, wireless transmission introduces further constraints, including added energy consumption, bandwidth limitations, and potential EMI. Addressing these issues requires low-power circuit design, efficient multiplexing, and optimized wireless integration. One possible solution comes from the field of neuromorphic and biomimetic event-driven design, where power and communication needs are regulated on demand.

The literature has increasingly focused on creating low-power interface circuits by minimizing hardware complexity [190]. Specifically, Kokolanski et al. [191] proposed a direct inductive sensor-to-microcontroller interface that completely eliminates power-hungry analog conditioning blocks. By connecting the sensor directly to a microcontroller using only one external resistor and a reference inductor, the system measures the discharging time constants of the network via digital I/O pins and internal hardware timers. This time-to-digital approach enables an onboard ratiometric calibration that automatically cancels out drift, achieving a non-linearity error under 0.3% full-scale span while maintaining an exceptionally low power profile. A recent study by Maru et al. [189] presents an inductive sensing system-on-chip for high-resolution (1 mm) sensing, which holds great potential in HMI. Compared to other works, the sensor achieves a minimum sensor voltage of 14 mV to 140 mV while consuming 124 μW at a 100 Hz scan rate. The system demonstrates submicrometer resolution, 25× faster acquisition, and 5× better energy efficiency.

Another concern is the aging of materials. In this regard, the most crucial element is the deformable layer, which is made of elastomers and polymers. Therefore, like other soft sensors, inductive ones are prone to viscoelastic effects, such as creep, stress relaxation, and hysteresis, which can lead to signal drift and degraded repeatability under prolonged or cyclic loading. This motivated the development of new materials and fabrication strategies to improve the mechanical performance, thus the repeatability of the sensor’s response. Nevertheless, as discussed before, the choice of fabrication method and material must consider the trade-off between final properties and ease of fabrication. In this context, 3D printing is a key tool for easily fabricating and tuning the sensor’s deformable layer on demand. However, there are strict limits on the printing parameters of the deformable layer, such as cell size and cell density, for these lattice structures.

Wearable devices and soft actuators inherently exhibit significant non-linearity due to complex geometries, hyperelastic material properties, variable loading conditions, and environmental fluctuations. Consequently, individual sensors require continuous calibration to maintain measurement fidelity [192]. High-density and multimodal soft mechanical sensor arrays suffer from significant cross-talk and mutual signal coupling. Without advanced decoupling algorithms and interference mitigation, this cross-channel degradation compromises data reliability and severely limits their practical utility in real-world wearable and robotic applications.

Significant improvements in the performance, reliability, and cost of smart SMI sensors can be achieved by simplifying their fabrication workflows. In particular, reducing the number of fabrication and assembly steps through single-step or integrated manufacturing approaches would greatly benefit sensor-integrated soft robots and wearable devices. Additive manufacturing techniques, such as multimaterial 3D printing, offer a promising route to directly embed inductive sensors, actuators, and conductive interconnects within soft structures. However, realizing such integrated systems requires advances in multimaterial printing processes and the development of compatible soft, highly conductive, and functional materials. These challenges highlight important research opportunities in the design of high-performance materials and in scalable multimaterial additive manufacturing for SMI sensors.

## Data Availability

The numerical and experimental data sets generated and analyzed during the current study are available from the corresponding author on reasonable request.

## Abbreviations

The following abbreviations are used in this manuscript:

SMI	soft mechanical inductive
EMI	electromagnetic interference
AC	alternating current
PCB	printed circuit board
CMOS	complementary metal-oxide-semiconductor
QF	quality factor
3D	three-dimensional
WPT	wireless power transfer
PDMS	polydimethylsiloxane
BCC	body-centered cubic
TPU	thermoplastic polyurethane
MRE	magnetorheological elastomers
NFC	near field communication
RF	radio frequency
FR4	flame retardant 4
FPC	flexible printed circuit
PI	polyimide
TSMC	Taiwan Semiconductor Manufacturing Company
MHD-LMC	multilayer high-density liquid-metal coil
HD-LMC	high-density liquid-metal coil
SIAS	soft inductive angle sensing
CNC	Computer Numerical Control
AR	aspect ratio
IMTS	inductive multimodal tactile sensor
AI-LMS	anisotropic inductive liquid-metal sensor
MC-PIS	magnetic crack-based piezoinductive sensor
FWMFS	flexible wide-range multidimensional force sensor
HMI	human–machine interfaces
